# Assessing Open-Ended Human-Computer Collaboration Systems: Applying a Hallmarks Approach

**DOI:** 10.3389/frai.2021.670009

**Published:** 2021-10-18

**Authors:** Robyn Kozierok, John Aberdeen, Cheryl Clark, Christopher Garay, Bradley Goodman, Tonia Korves, Lynette Hirschman, Patricia L. McDermott, Matthew W. Peterson

**Affiliations:** ^1^ The MITRE Corporation, Bedford, MA, United States; ^2^ The MITRE Corporation, Colorado Springs, CO, United States

**Keywords:** evaluation, assessment, human-machine teaming, collaborative assistants, multimodal, dialogue

## Abstract

There is a growing desire to create computer systems that can collaborate with humans on complex, open-ended activities. These activities typically have no set completion criteria and frequently involve multimodal communication, extensive world knowledge, creativity, and building structures or compositions through multiple steps. Because these systems differ from question and answer (Q&A) systems, chatbots, and simple task-oriented assistants, new methods for evaluating such collaborative computer systems are needed. Here, we present a set of criteria for evaluating these systems, called *Hallmarks of Human-Machine Collaboration*. The Hallmarks build on the success of heuristic evaluation used by the user interface community and past evaluation techniques used in the spoken language and chatbot communities. They consist of observable characteristics indicative of successful collaborative communication, grouped into eight high-level properties: robustness; habitability; mutual contribution of meaningful content; context-awareness; consistent human engagement; provision of rationale; use of elementary concepts to teach and learn new concepts; and successful collaboration. We present examples of how we used these Hallmarks in the DARPA Communicating with Computers (CwC) program to evaluate diverse activities, including story and music generation, interactive building with blocks, and exploration of molecular mechanisms in cancer. We used the Hallmarks as guides for developers and as diagnostics, assessing systems with the Hallmarks to identify strengths and opportunities for improvement using logs from user studies, surveying the human partner, third-party review of creative products, and direct tests. Informal feedback from CwC technology developers indicates that the use of the Hallmarks for program evaluation helped guide development. The Hallmarks also made it possible to identify areas of progress and major gaps in developing systems where the machine is an equal, creative partner.

## Introduction

Recent years have seen the rise of collaborative assistants such as chatbots, voice-activated assistants, and digital interfaces designed to answer questions and assist with relatively simple tasks (Ammari et al., 2019; Adamopoulou and Moussiades 2020). A next frontier is to create systems that more nearly approach Licklider’s 1960 man-computer symbiosis notion where one might “think in interaction with a computer in the same way that you think with a colleague whose competence supplements your own” (Licklider 1960). Such systems would be *collaborative machine partners* that contribute to complex, shared projects producing creative works and problem-solving. Collaborative partners require capabilities beyond those of most contemporary collaborative assistants, including “mutual goal understanding, preemptive task co-management, and shared progress tracking” (Bellamy et al., 2017). They also may require a model of the human’s abilities, intentions, and beliefs, and the ability to use nonverbal communication modalities (Lesh et al., 2004). In addition, for complex projects that require building with multiple steps, substantial ability to perceive, interpret, and refer to shared context is needed (Cohen 2017; Schaefer et al., 2019). The important contextual features can vary substantially according to the type of project. In a story generation scenario, context includes things like characters and their motivations, and previous story events. In a block-building activity, the task context involves the blocks and building surfaces and their locations. Finally, collaborative partners need to apply world knowledge and common sense (Bosselut et al., 2021; Hwang et al., 2020).

As researchers strive to create collaborative machine partners, criteria and methods for assessing them are needed to support development. For this, assessment approaches developed for other types of collaborative assistants that share properties could be adapted. Like collaborative machine partners, task-based dialog systems are designed for achieving particular goals; task-based dialog systems have often been evaluated with metrics that combine task success, dialogue efficiency, and dialogue quality (Walker et al., 1997). Pleasant engagement is important for collaborative machine partners as it is for chatbots; chatbots have recently been assessed in user studies for dialogue properties such as repetitiveness, interestingness, engagingness, and humanness (See et al., 2019). Ease-of-use is important for collaborative machine partners as well; usability heuristics have been created for user interfaces (Nielsen and Molich 1990) and more recently voice-activated assistants (Wei and Landay 2018; Zwakman et al., 2021). These usability heuristics serve as guidelines for development and are used in heuristic evaluation by usability experts to identify problems. While elements of these assessment approaches can be borrowed and combined, additional criteria are needed for collaborative machine partners’ more unique aspects, such as for greater use of various kinds of context (Brézillon 1996; Abowd et al., 1999; Oh, Schmidt, and Woo 2007; Jain et al., 2018; Böck and Wrede 2019), multiple communication modalities (Malchanau et al., 2019), and the generation of end-products that are often creative, complex, and lack a priori correct answers or even objective measures of completion (Hashimoto et al., 2019).

We present a set of criteria and methods for assessing collaborative machine partners. We propose eight properties that successful collaborative machine partners should have, and an associated set of observable features called the *Hallmarks of Human-Machine Collaboration*. The Hallmarks are intended to serve as guides for developers, diagnostics for development, and assessment criteria in evaluation. We outline methods for using the Hallmarks in direct tests by evaluators and in user studies and present examples of how we used the Hallmarks to assess a broad range of collaborative machine partners, including machine systems for story writing, building structures out of blocks, composing music, and exploring cancer molecular biology.

## Hallmarks Methodology

The Hallmarks approach focuses on properties of successful dialogue systems (*Key Properties*) and instances of those properties (*Hallmarks*) that can be observed or measured by evaluators. This approach relies on human judgment; it is not meant to be applied as an automated approach. The evaluation can be carried out by system developers or a neutral third party based on information collected about the interaction between the system and users; this information can be collected through instrumentation, observation, and surveys of the users. Evaluation involves analyzing the capabilities exhibited by each dialogue system during the interaction with users to determine which Hallmarks are met.

To define the Key Properties, we first identified characteristics of a system that could collaborate with humans in the way a colleague would. Our approach was pragmatic and driven by the varied needs of the kinds of collaborations in the program. We considered characteristics previously identified as being important in other collaborative assistants and used in evaluations, including in speech recognition and task-based spoken dialogue systems (Walker et al., 1997; Walker et al., 2000; Furui 2007), general heuristic approaches for the evaluation of human-machine interfaces (Molich and Nielsen 1990), and the notion of a habitable language for human-machine communication (Watt 1968), as well as drawing on our collective experience with chatbots, voice-activated assistants, and various other collaborative computer systems developed in research laboratories. For information about criteria and evaluations used for various collaborative assistant systems in the past and recently, see the *Literature Review* section. That section also includes a survey of several concurrently developed methods for the evaluation of multimodal dialogue systems (Venkatesh et al., 2018; Wei and Landay, 2018; See et al., 2019; Amershi et al., 2019; Malchanau et al., 2019) that did not factor into the development of the Hallmarks but have commonalities with our approach. We review similarities and key differences between these approaches and our own in the *Discussion* section of this study.

### Key Properties of Human-Machine Collaboration

As we defined our eight Key Properties of a successful collaborative machine partner, we further divided them into subcategories, as delineated below. Each Key Property is followed by a two-letter abbreviation we use to label the Hallmarks aligned to the property and a brief definition. The subcategories are then provided in a bulleted list under each Key Property and its definition.

**Successful Collaboration (SC)**: Satisfying creative collaborations can take place in which machines are not merely receivers of instructions but are full collaborators.

• ***Efficient, collaborative project completion***
• ***Worthwhile collaboration***
• ***Human satisfaction***

**Robustness (RO):** Efficient task-based interaction proceeds smoothly as long as the human wants to, without resets.

• ***Software reliability and consistency***
• ***Ability of human and machine to understand diverse communications***
• ***Ability of the machine to move the conversation forward past misunderstandings***

**Mutual Contribution of Meaningful Content (MC):** Each participant makes meaningful contributions to the session, and either party can take or cede initiative.

• ***Machine’s knowledge of when to act and how much to contribute***
• ***Appropriate and collaborative contributions***

**Consistent Human Engagement (HE):** Humans find engaging with machine comfortable, useful, fun, inspiring, and/or rewarding.

• ***Comfortable interaction***
• ***Machine’s ability to evoke and inspire***

**Context-awareness (CA):** Both partners can communicate efficiently by referencing and understanding contexts, including the linguistic, conversational, and deictic context, task context, goal context, self-knowledge, the partner’s abilities, and world/domain knowledge.

• ***Linguistic and/or deictic context-awareness***
• ***Pragmatic context-awareness***
• ***Situational context-awareness***
• ***Appropriate use of world/domain knowledge***

**Provision of Rationale (RA):** The machine can expose its reasoning, sources, and methods.

• ***Logging***
• ***Ability to explain rationale***
• ***Human***’ ***s trust is appropriately calibrated***

**Habitability (HA):** Humans easily learn to use language and/or gestures that the machine can correctly interpret and act upon.

• ***Shaping***
• ***Learnability***

**Use of Elementary Concepts to Teach and Learn New Concepts (EC)** ^1^: Uses and composes elements of a set of elementary concepts to represent more complex concepts.

• ***Representation***
• ***Composition***

These Key Properties and subcategories are multi-faceted; so to assess how well systems embody these properties, we identified observable features that a system succeeding in these dimensions would exhibit. These observables are our assessment Hallmarks. We provide some illustrative examples below. The full list of Hallmarks can be found in Supplementary Table 1 and is described in detail in the authors’ technical report (Kozierok et al., 2021).

For example, the ***Machine*** *’* ***s knowledge of when to act and how much to contribute*** subcategory of the **Mutual Contribution of Meaningful Content** Key Property reflects the goal of a collaborative machine partner participating in the bidirectional communication between humans and computers in which machines are not merely receivers of instructions but full collaborators.^2^ The observable Hallmarks we look for to determine how well that is being achieved include the following:

MC-1. Partners each take multiple turns in the interaction
MC-2. Each partner knows when to communicate and/or take actions
MC-3. Machine responses are of an appropriate length and level of detail
MC-4. The machine takes initiative when appropriate
MC-5. If the human grants autonomy, the machine responds appropriately

A completely successful collaborative machine partner should embody all subcategories of each of the Key Properties and satisfy all the Hallmarks. However, building a system that demonstrates all of the Key Properties, subcategories, and Hallmarks is an aspiration that today’s technologies may not be ready to attain. The Hallmarks framework can help a research team situate their work within the space of goal properties and allows evaluators to assess partial successes and trade-offs.

One exception is that under the ***Worthwhile collaboration*** subcategory of the high-level **Successful Collaboration** Key Property, we provide three Hallmarks that represent different ways of achieving that goal:

SC-3. It’s easier to do the activity together than alone
SC-4. Doing the activity together results in a more interesting, creative, or otherwise better product
SC-5. It is more enjoyable to do the activity together than alone

To achieve ***Worthwhile collaboration***, a system must demonstrate at least one of these three Hallmarks; typically, we do not see or expect to see all three for any given system. Different types of collaborative machine partners make the collaboration worthwhile in different ways. For example, a molecular biology system might make the collaboration worthwhile by providing information in ways that are *easier* to access than through other means, composing with a collaborative music system might be more *enjoyable* than doing it alone, and writing with a collaborative story writing system might result in a *more creative* story than writing alone.

It is also possible for different systems to achieve a single Hallmark in different ways. For example, to demonstrate Hallmark RO-11: *The machine copes with errors in the human’s input*; the system might take one of several actions, such as: make a guess as to the user’s intent and proceed, offer a useful error message (such as indicating which word it didn’t understand), or provide more structured shaping (offering examples of similar utterances that it can interpret). Evaluations of a system’s achievement of a Hallmark need not differentiate between alternate ways of achieving it.

Evaluation of a system using the Hallmarks is intended to provide feedback to the developer as to the system’s strengths and opportunities for improvement. It is not meant to provide a single score. Since the different Hallmarks are not all equally valuable, and their relative value varies according to the needs of the collaboration, it would not be meaningful to provide a score by counting the number of Hallmarks achieved or by computing the percentage of the Hallmarks in a subcategory that has been demonstrated. Additionally, it is beneficial to assess how well a Hallmark has been achieved — a system that achieves a subset of the Hallmarks especially well may have more real-life utility than one that barely checks off a larger set.

Some examples may be helpful to illustrate some of the other Hallmarks from our list. Note that the examples given below are not intended to convey the current capability of a system but to illustrate exemplars of successes, or Hallmarks noted as not yet satisfied during the development process. Some examples come from systems developed in the DARPA Communicating with Computers (CwC) program, whereas others are notional.

**FIGURE 1:**
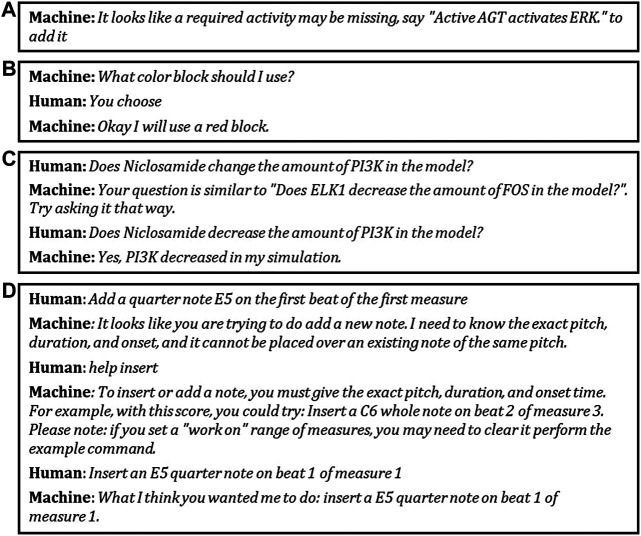
Initiative, autonomy, and shaping: Machine partners **(A)** take initiative, **(B)** accept offered autonomy, and in **(C)** and **(D)** help shape the human partners’ utterances. As described in the text, these examples demonstrate Hallmarks MC-4, MC-5, HA-1, and HA-2. The text for panel **(A)** was provided by the Bob with Bioagents system. The text for panel **(B)** was provided by Paul Cohen (Paul Cohen, personal communication, 2017). The text for panel **(C)** was provided by the CLARE system. The text for panel **(D)** was provided by the MUSICA system.

Figure 1 illustrates two related **Mutual Contribution of Meaningful Content** Hallmarks and shows two different CwC systems demonstrating **Habitability**: ***Shaping*** Hallmarks. In panel (A), the Bob with Bioagents system (Harvard Medical School, 2020) demonstrates Hallmark MC-4: *The machine takes initiative when appropriate* by pointing out something the human partner may have forgotten to specify. In panel (B), the human partner grants autonomy (allowing the machine partner to choose the block color), and the machine partner demonstrates Hallmark MC-5: *If the human grants autonomy, the machine responds appropriately* by choosing a color. In panels (C) and (D), we see the CLARE (Harvard Program in Therapeutic Science, 2020) and MUSICA (Stevens Institute of Technology, 2020) systems, respectively, demonstrating shaping. In each case when the human partner presents an input that the machine partner does not understand, the system suggests ways to modify the input to something they can move forward to advance the human partner’s goals. These interactions demonstrate Hallmarks HA-1: *The machine offers information that helps human partners produce utterances and/or gestures that the machine understands and is consistent with its capabilities* and HA-2: *The machine offers information that helps human partners produce utterances and/or gestures that are consistent with the partners’ goals and move the collaborative effort forward.*

**FIGURE 2:**
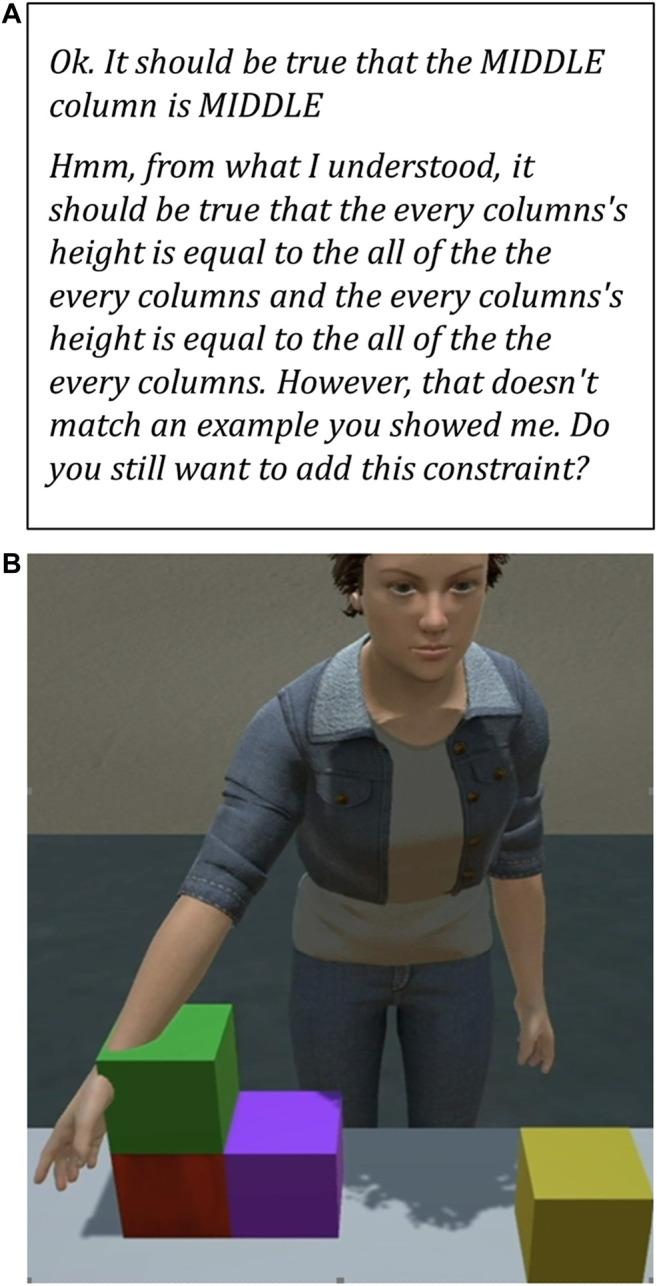
Undue distraction: Early versions of two different CwC systems demonstrate negative examples of Hallmark HE-4: *The machine communicates without creating undue distraction*. **(A)** A distracting (hard to understand) machine-generated description of a constraint learned by an early version of the CABOT system (Perera et al., 2018). **(B)** A distracting (physically impossible) machine-generated visualization in an early version of the Diana system (Pustejovsky 2018).

Figure 2 gives two negative examples of the **Human Engagement** Hallmark HE-4: *The machine communicates without creating undue distraction.* In panel (A), we see a text description of a constraint that is so hard to understand as to be distracting. In panel (B), we see an avatar reaching through a block, distracting from the interaction with the physical impossibility of the action.

**FIGURE 3:**
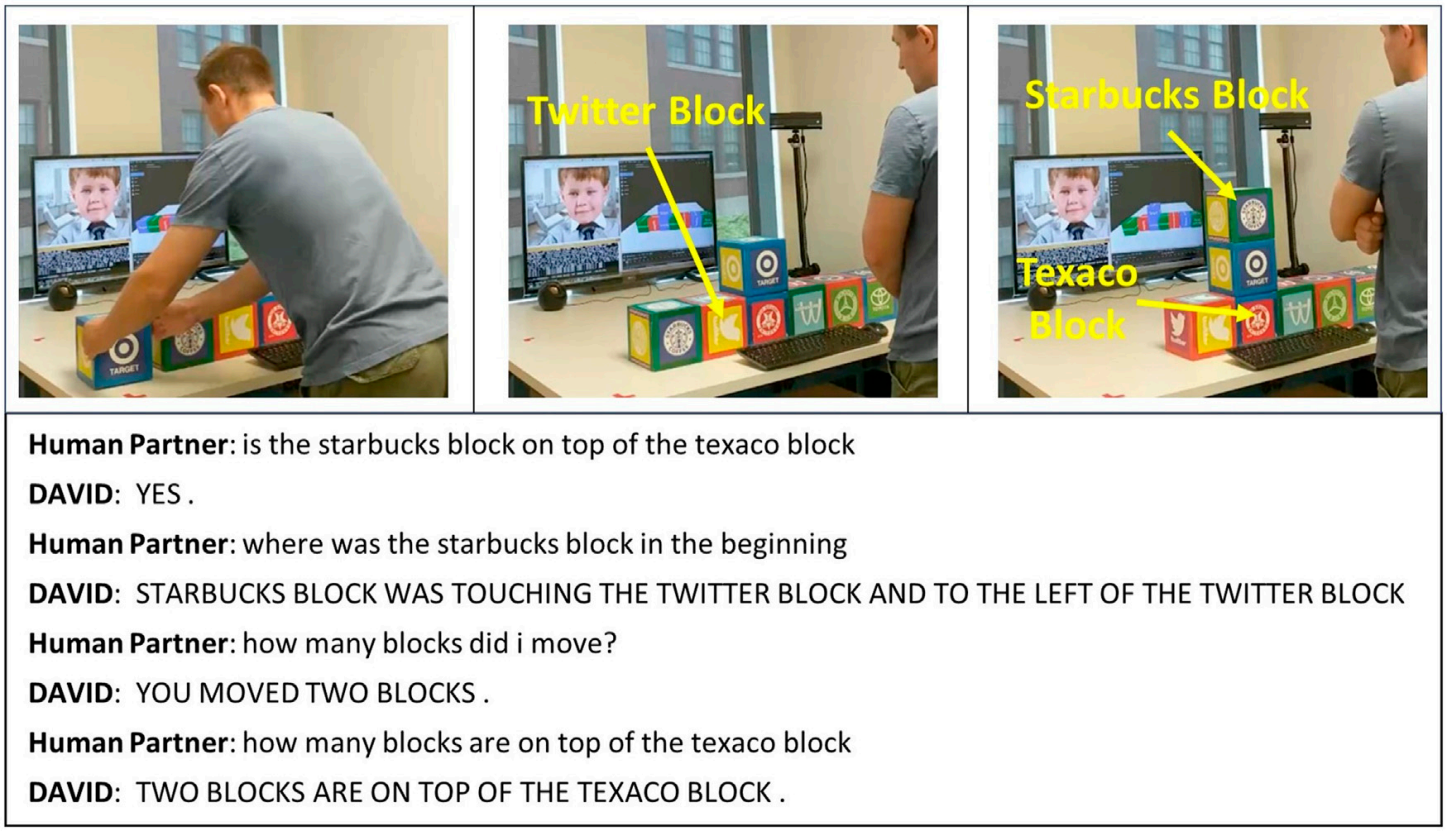
Awareness of the evolving situation: DAVID is an agent that works with a human user partner to manipulate blocks and answer spatial and temporal questions about the evolving blocks world scene. Here the system demonstrates **Context-awareness** Hallmark CA-14: *The machine responds appropriately to human references and actions in the context of the evolving situation* (*includes anything built and pieces available*) by correctly answering questions about the locations of blocks both before and after the block moves shown. This dialogue takes place at the time of the third photo. The human partner’s utterances are spoken, and the log excerpt here shows the computer’s interpretation of the speech. The images and associated log text were provided by the DAVID system (Georgiy Platonov, unpublished data, 2020).

Figure 3 gives an example of the DAVID system (Platonov et al., 2020) demonstrating awareness of the evolving scene by correctly answering questions about the locations of blocks both before and after the human partner moves some of the blocks. This satisfies the ***Situational context-awareness*** Hallmark CA-14: *The machine responds appropriately to human references and actions in the context of the evolving situation (includes anything built and pieces available)*.

**FIGURE 4:**
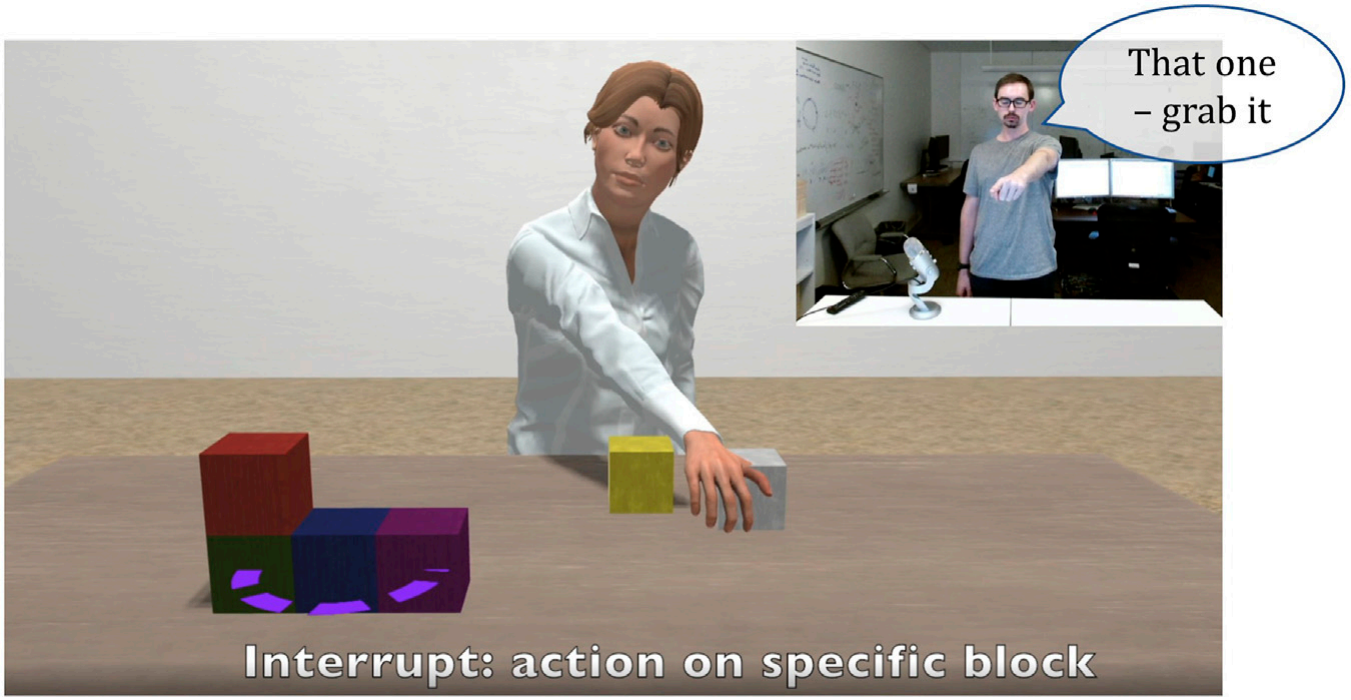
Multi-modal interaction with asynchrony: As Diana reaches for the white block, the human partner interrupts her by pointing to the blue one and asking her to grab it instead. (The human is pointing from his perspective at the place on the virtual table indicated by the purple outline.) As described in the text, this example demonstrates Hallmarks RO-9, CA-6, MC-2, HE-1, and HE-2. The image was provided by the Brandeis Diana team (Krishnaswamy and Pustejovsky, email to the authors, February 5, 2021).

Figure 4 shows the Diana system (Krishnaswamy and Pustejovsky, 2018; Krishnaswamy 2020) demonstrating multiple Hallmarks across four Key Properties. In this example of multimodal interaction with asynchrony, the human partner interrupts Diana as she reaches for one block and instructs her to grab a different one instead, indicating the correct block with a pointing gesture. The Diana system correctly interprets the speech coupled with the pointing gesture, demonstrating Hallmark RO-9: *The machine correctly interprets multiple communication modalities*. In addition to aligning the gesture with the corresponding spoken command, the machine must also correctly interpret the deictic (in this case, spatial) context of the gesture demonstrating Hallmark CA-6: *The machine correctly interprets and correctly uses deictic references (i.e., references situated in time and/or place, such as by pointing).* The ability to interrupt Diana to correct a misunderstanding or simply to change the original command provides very fluid interactions between Diana and the human partner, demonstrating Hallmarks MC-2: *Each partner knows when to communicate and/or take actions*, HE-1: *Human partners can communicate successfully in a way that is comfortable,* and HE-2: The *human is satisfied with the pacing/tempo of the interaction.*

**FIGURE 5:**
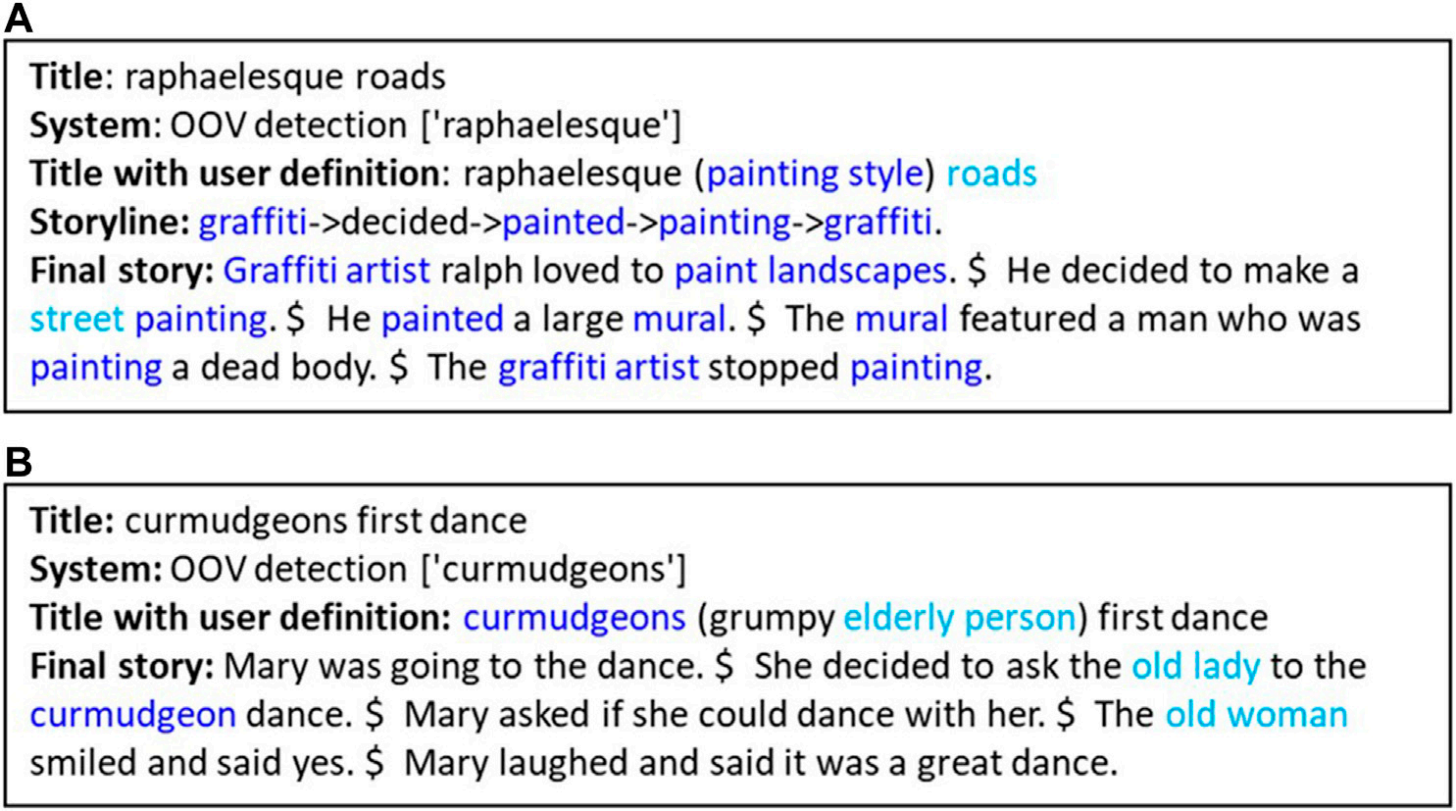
Out-of-vocabulary (OOV) detection and management: As described in the text, these examples show a collaborative story writing system dealing with out-of-vocabulary terminology, demonstrating Hallmarks CA-4, CA-5, and CA-9. The examples were provided by the ISI human-computer collaborative storytelling system (Goldfarb-Tarrant et al., 2019; Yao et al., 2019).

Figure 5 shows a collaborative story writing system demonstrating three Context-awareness Hallmarks in dealing with out-of-vocabulary terminology. In both panels, the system detects that a term is out-of-vocabulary (OOV detection) demonstrating Hallmark CA-9: *The machine indicates that it doesn’t understand what a particular entity/action/word/gesture is when appropriate*. In the user interface (not shown), the human partner is asked to provide a brief definition which we see in both panels in parentheses in the “Title with user definition” line. In panel (A), the system incorporates terms related to the human partner’s definition of the OOV term into the storyline and story. Other terms in the title also influence the story (turquoise). In panel (B), the system incorporates a form of the OOV (blue) and elements of the human’s definition (turquoise) into the story it generates. Both examples demonstrate Hallmarks CA-4: *The machine correctly interprets a term defined by a human partner*, and CA-5: The *machine uses a term defined by a human partner*.

**FIGURE 6:**
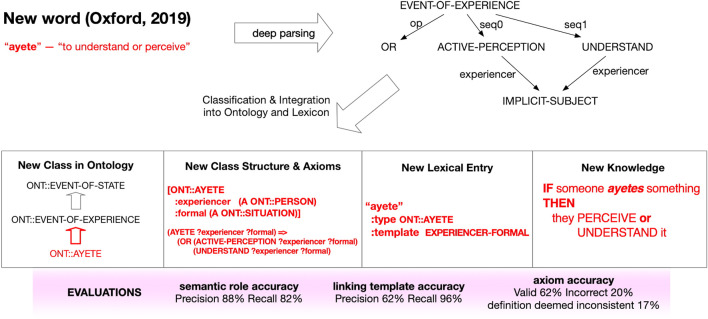
Building new concepts from known concepts: When the TRIPS system encounters an unknown word, it is able to look the word up in a dictionary, and based on the definition, it extends its ontology with a new concept, including axioms that relate the new concept to existing concepts and build lexical entries for the word that enables sentences including the new word to be understood. The figure shows how the word “ayete”, listed as a new word in the Oxford dictionary in 2019, is processed, adding the new understanding that represents the new word “ayete” in terms of known concepts “perceive” and “understand”. This demonstrates Hallmark EC-3. The image was provided by IHMC (James Allen, email to the authors, February 5, 2021) to illustrate how this capability works.

Figure 6 shows how the TRIPS system (Allen et al., 2020) can learn the meaning of a new word without human intervention, satisfying Hallmark EC-3: *The machine can learn (or infer) the meaning of a new word or concept without explicit human instruction* under **Use of Elementary Concepts to Teach and Learn New Concepts.** When TRIPS encounters an unknown word, it is able to look the word up in a dictionary and add that word to its vocabulary. The system performs a deep understanding of language, mapping English sentences to a formal knowledge representation supporting reasoning. This requires knowledge about both a word’s meaning (semantics) and the structural role it plays in sentences (syntax).

### Uses of the Hallmarks in Development and Assessment

The Hallmarks can serve as 1) guidelines for developers, 2) criteria for assessing progress in developing machine capabilities, and 3) heuristics for identifying opportunities for improvement in systems. The Hallmarks are not intended to be a checklist, where every Hallmark must be observed for a system to be considered successful. Rather, the breadth of the Hallmarks offers alternative means by which a broad range of systems can be developed and evaluated for the Key Properties. These Hallmarks and Key Properties were first developed in the context of the DARPA CwC program, for which the authors served as the Test and Evaluation (T&E) team. In the CwC program, research teams often chose a subset of Hallmarks to develop capabilities around, and evaluations then assessed progress on the chosen Hallmarks. In turn, heuristic-style evaluations using the Hallmarks identified improvements that could be useful to users and influenced the selection of new Hallmark-related capabilities to develop. Nearly all systems were assessed with a combination of two general methods: direct use and assessment by evaluators, and user studies in which appropriate test users interacted with the systems.

#### Direct Use and Assessment by Evaluators

The authors, in our T&E role, directly interacted with each system and evaluated each with the Hallmarks based on their direct experience and by analyzing logs or recordings of their sessions. These evaluations consisted of short sessions with one to three evaluators and were conducted multiple times over the course of development for each system. In one form of assessment, MITRE T&E team members acted as human partners attempting to accomplish goals and trying various ways of communicating. These interactions were analyzed to identify instances where the system exhibited particular Hallmarks; frequently, these analyses focused on Hallmarks that were particularly important at the stage of development or related to the development team’s research focus areas. In addition, these interactions were also used to conduct heuristic evaluations, where the evaluators identified problems and associated Hallmarks to inform further development. In a second form of assessment, the evaluators assessed specific capabilities associated with particular Hallmarks by creating and running a set of test utterances. The Hallmarks and specific capabilities for testing were selected by the MITRE T&E team and the CwC research teams based on likely importance to system usability or to assess progress towards the teams’ specific Hallmark-related goals.

#### User Studies

User studies were used to assess whether systems successfully exhibited Hallmarks in the intended user context, as well as to identify impactful opportunities for improvement. The user studies were designed and conducted by the researcher teams with input from the MITRE T&E team, and the T&E team evaluated the resulting study data. In most cases, the specific activities that human partners would be asked to work on were developed by the T&E team, in order to come up with activities that were consistent with the evaluation goals and the capabilities of the system at the time of the user test, without being known to the researchers during development. CwC research teams obtained Institutional Review Board approval from their own institutions as needed. User study participants were required to have little to no prior experience with the systems. In addition, for the music and molecular biology systems, participants were required to have appropriate domain expertise. For user studies involving lengthy sessions, the number of participants was often less than ten due to the time intensiveness for conducting the user studies and manual evaluation of the study data with the Hallmarks. For each user study, we analyzed one or more of the following types of data: direct observations of participants interacting with systems, either live or recorded; logs of participant-system interactions; participant survey responses; and third-party evaluations of the products of participant-system collaborations. Multiple types of data were typically used because different types of data are required for assessing various Hallmarks.

#### Log Analyses

Most of the Hallmarks can be assessed with direct observations of human-machine interactions or logs of these interactions. Consequently, we requested human-readable logs of human participant-system interactions for all systems. These logs included timestamped human and machine utterances, gestures, and actions with simultaneous information about the status of the shared environment and structures, models, compositions, or stories being created.

Some of the Hallmarks can be observed in logs at the level of a single human-machine exchange, consisting of a human utterance, gesture, or action, and the corresponding machine response. These Hallmarks can be assessed by calculating how frequently the Hallmark is achieved or missed in exchanges across logs. Examples include many under **Robustness** such as RO-6 *The machine handles multiple phrasings or forms for similar requests* and *RO-13 The machine provides helpful/actionable error messages*, as well as some under other properties such as **Provision of Rationale** RA-4 *The machine answers questions about its reasoning, sources, and/or methods*, and **Habitability** HA-4 *The machine answers questions about its capabilities.*

Other Hallmarks, particularly under the Property **Context-aware**, require examining information beyond that in a single exchange. Some require examining multiple, sometimes non-contiguous, exchanges in concert, such as CA-1 *The machine recognizes co-referring mentions of previously mentioned entities (co-reference resolution)*, CA-4 *The machine correctly interprets a term defined by a human partner*, and CA-12 *The machine tailors responses to the human partner’s stated or implied goals*. Some Hallmarks require examining exchanges in concert with information about the shared setting and things being built or composed, as in CA-14 *The machine responds appropriately to human references and actions in the context of the evolving situation* and CA-15 *The machine’s contributions to the interaction are consistent, relevant, and build from turn to turn*. Still, others sometimes require examining exchanges in concert with information from outside the system, such as CA-*16 The machine applies world/domain knowledge as required*.

We examined logs to assess whether systems successfully exhibited Hallmarks as well as to identify where Hallmarks were not yet achieved but could be beneficial. Typically, we assessed whether each machine response in a log was a correct or appropriate action, informative answer, or helpful suggestion, or was an incorrect, inappropriate, uninformative, or unhelpful response. We then identified the Hallmarks that contributed to the success of correct and helpful responses, and the Hallmarks that, if they had been achieved, could have prevented problems in incorrect and unhelpful responses. Typically, identification of Hallmarks associated with successful responses focused on Hallmarks that were developmental goals or of particular interest for a system, while identification of Hallmarks associated with problematic responses tended to use a broader set of Hallmarks to identify opportunities for improvement.

Other Hallmarks, such as those under the Property **Mutual Contribution of Meaningful Content** like MC-6 *The machine makes meaningful contributions to the interaction*, were assessed using a somewhat different type of log analysis, where each partner’s contributions to the interaction or final product were attributed and cataloged.

#### User Surveys and Third-Party Review of Creative Products

Hallmarks about the human participant’s experience generally cannot be assessed from logs. Instead, these can be assessed through Likert-scale survey questions (Likert 1932), which provide a way to solicit a person’s level of agreement or disagreement with a particular (Likert) statement. In the case of human-machine collaborative systems, they can be used to collect feedback on a human participant’s satisfaction with an aspect of the system, such as a particular system feature or ease of use of the system. For example, the Hallmark HE-1, *Human partners can communicate successfully in a way that is comfortable*, can be assessed with a Likert-scale survey item: *I was able to communicate successfully in a way that was comfortable*. We developed a standard set of Likert-scale survey questions to pose to human partners for each of the Hallmarks that need to be assessed in that way. When the research teams executed their user studies, they chose from among these questions, according to which Hallmarks they wanted to assess. In some cases, these were modified slightly during consultation between the research team and the T&E team to better reflect the details of the system being evaluated. The full list of standard survey questions mapped to Hallmarks can be found in Supplementary Table 1. *Evaluating Composition by Communication, a Music Composition Collaborator* section, includes an example of survey questions that were modified for the particular use case. Surveys also sometimes included open-response questions to collect additional Hallmark-related information.

Hallmarks about the quality, creativity, coherence, etc. of products created through collaboration can be assessed by having third parties review the products. This can be done *via* crowd-sourcing platforms such as Amazon Mechanical Turk. For example, the Hallmark SC-4, *Doing the task together results in a more interesting, creative, or otherwise better product*, can be assessed by having crowd-workers rate products created by human-system collaborations *versus* solely by human participants.

## Case Studies

We present examples of how the Hallmarks were applied to collaborative machine partners built for a variety of activities, including creating animated stories, building with blocks, story writing, composing music, and discovering molecular mechanisms. These cases here are not full evaluations of final systems but rather are illustrative examples of assessments at various stages of systems’ development during the DARPA CwC program.

### Assessing Robustness in an Early Version of a Visual Storytelling System

Visual storytelling is the process of building and conveying a narrative in a visual medium (as contrasted with storytelling in an auditory or textual medium). The goal in a human-machine collaborative system for visual storytelling is to build a *scene* populated with *characters* and then *animate* the characters in the scene to tell a story. The Aesop Visual Storytelling (Meo et al., 2019) system developed by SRI International integrates an animation system with a natural human interaction system using speech and text input to provide an environment for a human to collaborate with the system to design characters, establish sets, and create animations to produce short movies. The system produces voluminous logs of interactions, primarily for the purpose of developing and debugging what is a rather complex system. Initially, these logs were not human-readable.

**Robustness** Hallmarks were assessed after observing an interaction and manually recording the utterances given to the system, as well as noting the changes in the visual interface. This assessment permitted our team to give early feedback to SRI about the robustness of the Aesop system at the time, as well as to provide them with feedback about how to improve their logs to facilitate analysis.

**FIGURE 7:**
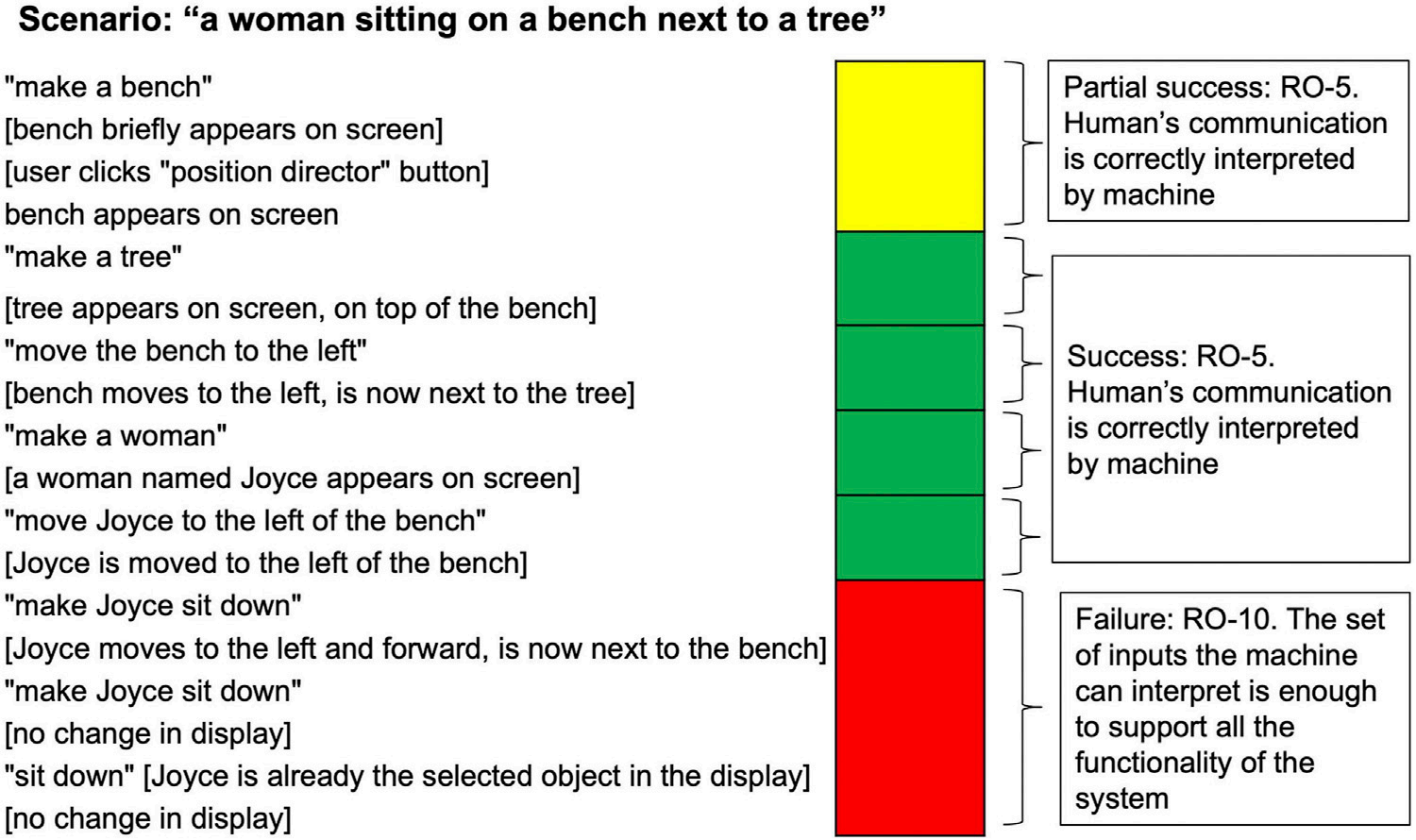
Robustness assessment of a simple interaction in the Aesop system: On the left of the figure are human utterances (in quotation marks) paired with descriptions (in square brackets) of the state of the screen where the characters and scene are being built. In addition to the Hallmarks annotated in the image, this interaction demonstrates an overall success on Hallmark RO-1.

Figure 7 shows a sample robustness assessment of a simple interaction in an early version of the Aesop system. The end goal of the interaction is “a woman sitting on a bench next to a tree”. In this early version of Aesop, there was no utterance the human could offer that would cause the character to sit down, resulting in the failure on Hallmark RO-10: *The set of inputs the machine can interpret is enough to support the full functionality of the system*. The first interaction is only considered a partial success on Hallmark RO-5: *The human’s communication is correctly interpreted by the machine* because the user had to resort to using the graphical user interface (“position director” button) instead of being able to complete the action through the dialogue system. The entire interaction depicted is deemed successful for RO-1: *The interaction proceeds without the need for resets,* though this Hallmark is usually assessed over the course of a longer session.

### Surveying Human Partners Following Use of a Blocks World System

The Blocks World use case re-visited a problem addressed by numerous AI researchers in the 1970s including the seminal natural language understanding and planning research of Winograd (1972) on the SHRDLU system. A human user could interact with SHRDLU using natural language text commands (e.g., “Pick up the red block”). The CwC Blocks World use case similarly included manipulating blocks on a table through collaboration with an AI-based agent but was broader in scope; for example, it included the challenge of teaching the agent a new block structure shape such as a pyramid. The example Blocks World system described here illustrates how the results from surveys of users of the system can be used to determine which and how well particular Hallmarks have been achieved.

**FIGURE 8:**
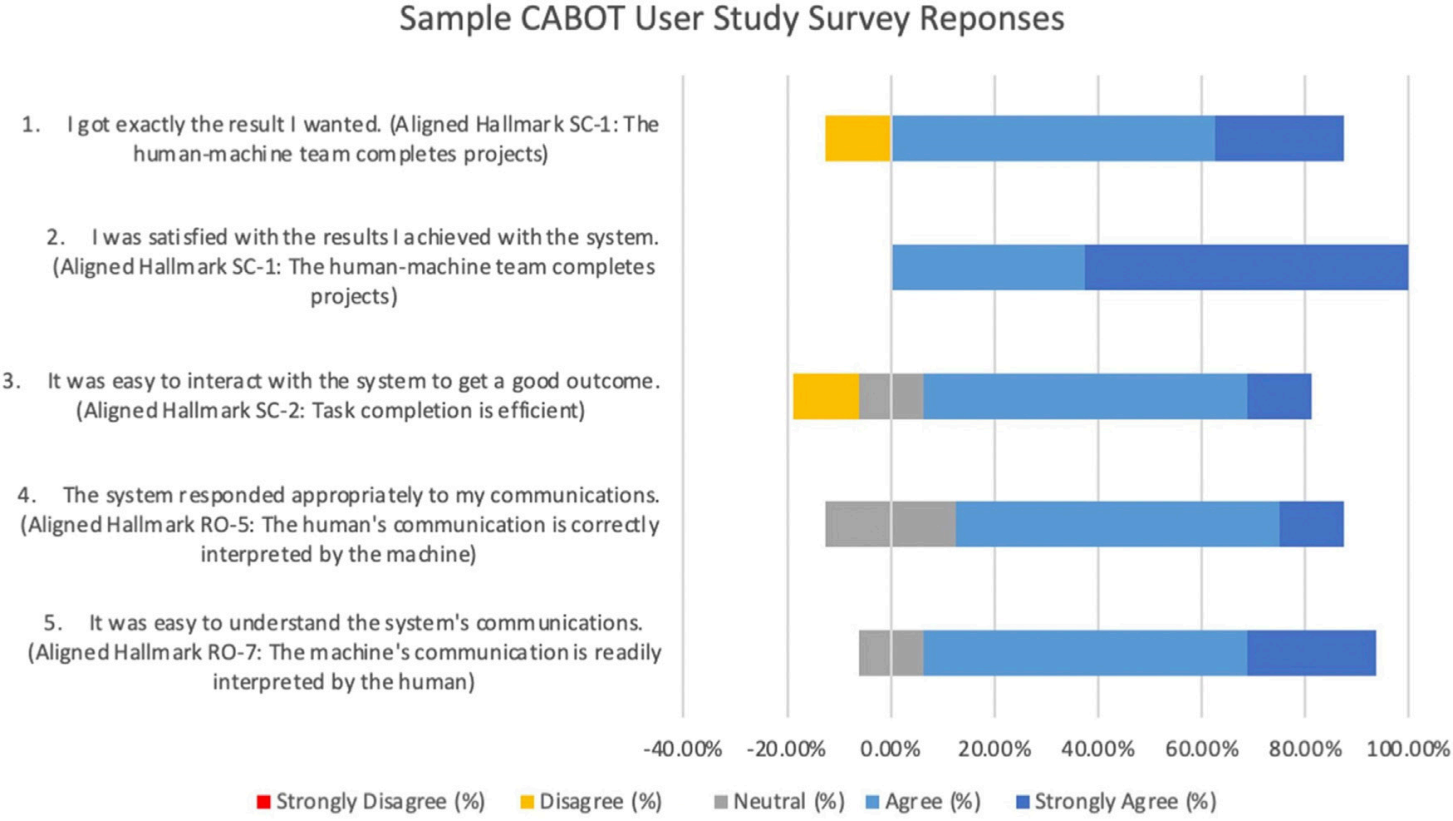
Sample chart generated by the authors from results of Participant Surveys from IHMC User Study (Ian Perera, unpublished data, July 2020) on CABOT Blocks World System (Perera et al., 2018). Survey items are shown along with their aligned Hallmarks and participant assessments. Eight individuals participated in this study.

The IHMC CABOT Blocks World System (Perera et al., 2018) allows a human partner to specify a set of constraints and examples to define a block structure shape (e.g., a “corner”) through a dialogue between the human and the system. The evaluation of this system was conducted by giving each human participant a set of block structure shapes to teach the system and then giving the participant a survey to fill out at the conclusion of their session with the system based on the standard survey questions provided by the MITRE T&E team. A sample of those results is shown in Figure 8 along with the Hallmarks aligned with each survey statement.

### Assessing Machine Contributions in Collaborative Story Writing Systems

Collaborative story writing allows human and machine partners to contribute sentences that create a story. CwC goals included interactions in which the machine contributions inspire the human to be more creative and stories whose machine and human contributions create a coherent result.

For one story writing system, machine contributions were assessed *via* third-party review. The University of Washington Collaborative Story Generation system provides a New Yorker cartoon as a prompt for a 10-sentence story. In an evaluation of an early system prototype, the human study participants performed a task in one of two conditions, alone (solo condition) or partnered with machine suggestions (machine-in-the-loop (MIL) condition).

The development team obtained third-party ratings of the output generated by their collaborative story generation system interactions *via* the crowd-sourcing platform Amazon Mechanical Turk. These ratings can be aligned with Hallmarks for assessment. Here is an example of Amazon crowd-worker ratings for the collaborative story generation system described in Clark et al. (2018):

“There was no statistically significant difference in the Amazon Mechanical Turk third-party evaluation ratings between the solo and MIL conditions. For creativity, the average score for the solo condition was 4.87 and was 4.84 for the MIL condition (*p* = 0.88).”

The rating of creativity (made on a 7-point scale) aligns with the **Successful Collaboration** Hallmark SC-4: *Doing the task together results in a more interesting, creative, or otherwise better product.* In this case, the evaluation did not demonstrate success on this Hallmark on this early prototype system, motivating the team to improve user satisfaction. A subsequent evaluation, though not strictly parallel, indicated greater user satisfaction with creativity, with ratings of 3.8 on a 5-point scale in response to the statement, “The suggestions helped me come up with new ideas.” (Elizabeth Clark, unpublished data, February 8, 2021).

For another story writing system, machine contributions were assessed *via* logs and human partner survey results. The Stanford *Writing with Artificial Intelligence* story generation system (Mina Lee, Chris Donahue, and Percy Liang, unpublished data, June 25, 2020) enables human partners to generate a story in response to one of four prompts presented by the system. The system suggests text that the human partner can select for incorporation into the story, and the suggestions are conditioned on the context surrounding the location of a human’s cursor. The system has an infilling strategy that permits users to backtrack and generate text for earlier parts of their stories as they see fit (Donahue, Lee, and Liang 2020).

We analyzed logs of user interactions to compute how much of the system’s suggestions were used in the final story and coupled that with results of the participant surveys. The content of the logs shows that users accept about 4% of the system’s suggestions and that only 11 (9%) of the 121 stories contain no system-suggested text. This indicates that the system made useful contributions to the stories (Hallmark MC-6: *The machine makes meaningful contributions to the interaction*).

**FIGURE 9:**
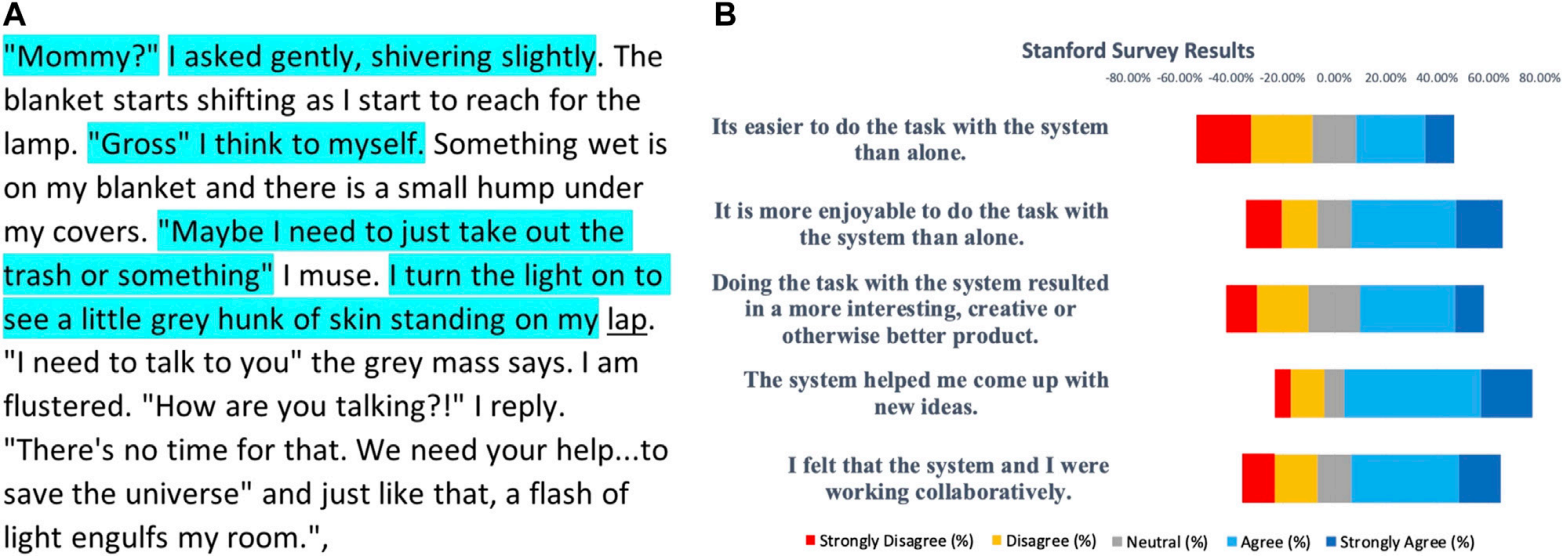
**(A)** Final story generated by the Writing with Artificial Intelligence system, where turquoise shading represents system suggestions selected by the human partner and present in the final story, and underlining indicates word substitution by the human partner. (Hallmark MC-6: *The machine makes meaningful contributions to the interaction.*) **(B)** Chart of participant survey responses.

Figure 9 panel (A) shows a story generated from the system and human contributions, where the human partner inserted text not only at the end of the existing text but also in between and within existing sentences. The sequence of suggestions was as follows:

• System suggests: “*Mommy?*”
• Human retains system’s suggestion.
• System suggests: *I asked gently, shivering slightly.*
• Human retains the system’s suggestion.
• Systems suggests: *Gross*“ *I think to myself* ”*Maybe I need to just take out the trash or something*“ *I turn the light on to see a little grey hunk of skin standing on my pillow.*”
• Human edits the system’s suggestion, adding *Something wet is on my blanket and there is a small hump under my covers* after the word *myself,* adding *I muse* after *something,* and replacing *pillow* with *lap.*
• Human then adds: “*I need to talk to you*” *the grey mass says. I am flustered.* “*How are you talking?!*” *I reply.* “*There*’*s no time for that. We need your help... to save the Universe*” *and just like that, a flash of light engulfs my room.*

Survey results also indicated that human partners believed that the system produced and inspired creative ideas and that working with the system resulted in a more interesting, creative, or otherwise better product. Out of 100 survey responses to the question “Doing the task with the system resulted in a more interesting, creative or otherwise better product,” (assessing SC-4: *Doing the task together results in a more interesting, creative, or otherwise better product*), 48 agreed or strongly agreed, 20 were neutral, and 32 disagreed or strongly disagreed. Out of 100 survey responses to the question “The system helped me come up with new ideas,” (assessing HE-5: *The machine produces content that is interesting, novel, useful, and/or creative*), 53 agreed or strongly agreed, 23 were neutral, and 24 disagreed or strongly disagreed. Out of 100 survey responses to “The system helped me come up with new ideas,” (assessing HE-6: *Machine inspires new ideas in the human partner*), 73 agreed or strongly agreed, 8 were neutral, and 19 disagreed or strongly disagreed (Chris Donahue, unpublished data, June 25, 2020). Additional survey results are shown in Figure 9 panel (B).

### Evaluating Composition by Communication, a Music Composition Collaborator

The MUSICA system (Stevens Institute of Technology, 2020) allows human partners to compose music using both natural language- and graphical user interface-based interaction with a musical score.^3^ Music composed and arranged by the user can be used as the basis for improvisational exchange between the user and the system as an automated backing band plays in the background. These capabilities are arranged into three distinct tasks: Generate (the creation of short musical segments), Organize (the arrangement of short musical segments into longer compositions), and Jam (interactive improvisation between the human and the machine). The goal of MUSICA is to make it easier for both musical novices and experienced musicians to compose pieces of music and to practice musical improvisation in a group setting.

The performance of the system was evaluated against the Hallmarks in two ways: 1) through the administration of a survey to human participants in user studies where the questions were designed to correspond to Hallmarks and 2) by examination of human-computer interaction logs provided to the MITRE T&E team to identify capabilities aligned with the Hallmarks. Both the responses to surveys and the interactions of T&E team members with the MUSICA system were used to assess the progress against Hallmark-related goals selected by the system’s developers.

Many of the survey items (which were assessed using a Likert scale) administered to study participants are mappable to Hallmarks. For example, the item “It was easy to interact with the system to get the intended outcome” may be mapped to Hallmark SC-2: *Task completion is efficient*. The item “I felt that the system and I were working collaboratively” may be mapped to Hallmark MC-6: *The machine makes meaningful contributions to the interaction*.

The MUSICA system also supports musical exchanges between the user and system in the form of improvisational jazz solos during the “Jam” task. The quality of these exchanges is also assessed through survey items aligned to Hallmarks. These include “The computer’s solos were creative”, which may be mapped to Hallmark HE-5: *The machine produces content that is interesting, novel, useful, and/or creative* and “The computer recognized my ideas/motifs” which may be mapped to Hallmark RO-5: *The human’s communication is correctly interpreted by the machine*. These represent task-specific variations of the standard Hallmark-aligned survey items. Teams were permitted to make such changes to the standard survey items with the approval of the T&E team.

**FIGURE 10:**
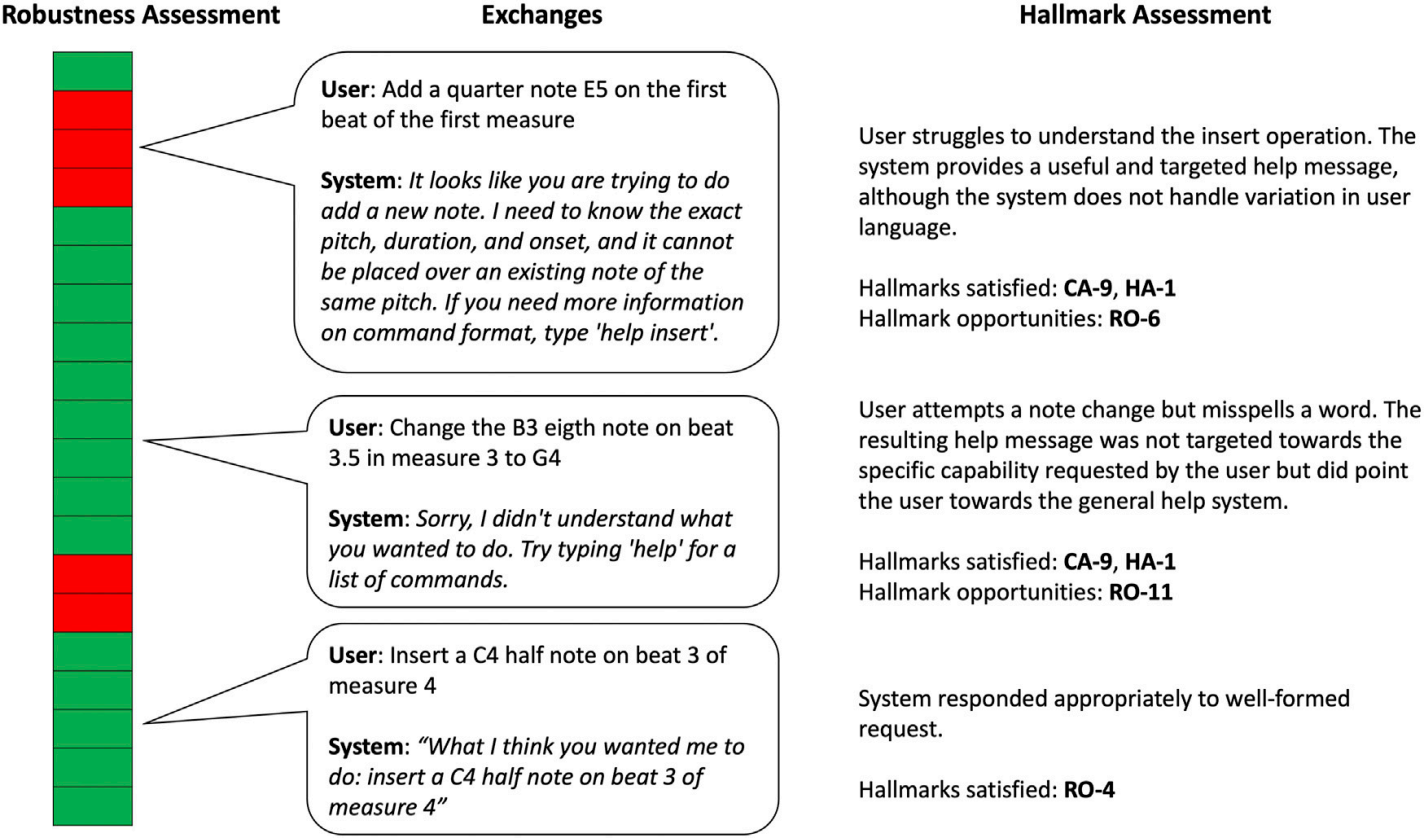
An example of the coding of a composition by conversation human-computer interaction log for robustness. Green blocks represent successful interactions between the human partner and the system. Red blocks indicate those interactions where the machine did not respond appropriately. Hallmarks satisfied by the exchange as well as Hallmarks that aren’t satisfied but might be potential opportunities for improvement are indicated under the “Hallmark Assessment” column.

In addition to assessing the MUSICA system against the Hallmarks through surveys of participants in user studies and through direct interaction with the system, the evaluation team also evaluated the robustness of the MUSICA system by annotating interactions in logs produced during user studies. Logs were analyzed for task completion as well as the success or failure of individual interactions. Figure 10 shows an example of one of these logs and includes annotations of the success of individual interactions. Several interactions have been highlighted along with the Hallmarks demonstrated by these interactions and Hallmarks that were not satisfied but might be good opportunities to improve system performance.

Based on these assessments, the MUSICA system was considered to be successful when measured against several Hallmarks, including RO-1: *The interaction proceeds without the need for resets (no crashes/hangs)*, RO-9: *The machine correctly interprets multiple communication modalities*, RO-13: *The machine provides helpful/actionable error messages*, HE-1: *Human partners can communicate successfully in a way that is comfortable*, and HA-1: *The machine offers information that helps human partners produce utterances and/or gestures that the machine understands and are consistent with its capabilities*.

### Identifying Hallmark Successes and Opportunities for Bob, a Molecular Biology Machine Collaborator

The goal of this collaboration is to discover new mechanisms, create explanations, and build molecular models about drug effects, cancer, and other biology. In this use case, a curious researcher with biological expertise collaborates with a machine that can offer information from many databases and millions of publications. To address this use case, a team of researchers from Harvard Medical School, Smart Information Flow Technologies (SIFT), Tufts University, Oregon Health & Science University, and The Florida Institute for Human & Machine Cognition (IHMC) built a molecular biology dialogue-based system called Bob with Bioagents (Harvard Medical School, 2020). Bob has ∼60 different biological information capabilities, including answering queries about various types of molecular interactions, displaying pathways and gene expression data, collaboratively building models, and running simulations.

One way that Hallmarks were used with Bob was in evaluating progress on Hallmark-aligned capabilities during Bob’s development with the use of test utterances. With the Bob development team, we identified specific Hallmark-related capabilities that could be useful to researchers if incorporated into Bob. For some Hallmarks, there were multiple specific capabilities. We assessed these capabilities periodically by creating test utterances and evaluating Bob’s responses. Typically, five to ten test utterances were used per specific capability to assess progress status. Table 1 shows a sample of the Hallmarks, specific capabilities aligned with these Hallmarks, test utterances, and examples of what we considered to be appropriate responses.

**TABLE 1:** Examples of Hallmark-related capabilities assessed with test utterances during Bob’s development.

Property & sub-cat	Hallmark	Specific capability	Example test utterance	Example appropriate response
Robustness: Understanding diverse communications	The machine handles multiple phrasings or forms for similar requests	Understand imperative form	Tell me if STAT3 is a transcription factor	Yes, it is
		Interpret alternative ways of asking a question	Can you tell me whether BRAF is a kinase?	Yes, it is
		Interpret queries that require performing two tasks to answer	What kinases does STAT3 regulate? <Requires identifying what STAT3 regulates and then which are kinases>	I found 53 kinases regulated by STAT3: <and list only kinases>
Robustness: the conversation forward past misunderstandings	The machine copes with errors in the human’s input	Cope with typos (not in entity names)	What drugs drugs target BRAF?	I found 10 drugs for BRAF: ALW-II-38–3, AZ-628, Dabrafenib, ...
		Cope with misspelled entities	What does selumitinibib target?	I interpreted “selumitinibib” as “selumetinib”. The nominal target of selumetinib is MAP2K1
	The machine asks clarifying questions as needed	Ask clarifying questions about ambiguous entities	What does ERK phosphorylate?	By ERK, do you mean ERK1 or ERK2?
Context-awareness: Pragmatic	The machine indicates that it doesn’t understand what a particular entity/action/word/ gesture is when appropriate	Indicate when it doesn’t know an entity	What drugs target XYZfakeprotein?	I couldn’t interpret “XYZfakeprotein” as a valid protein

**FIGURE 11:**
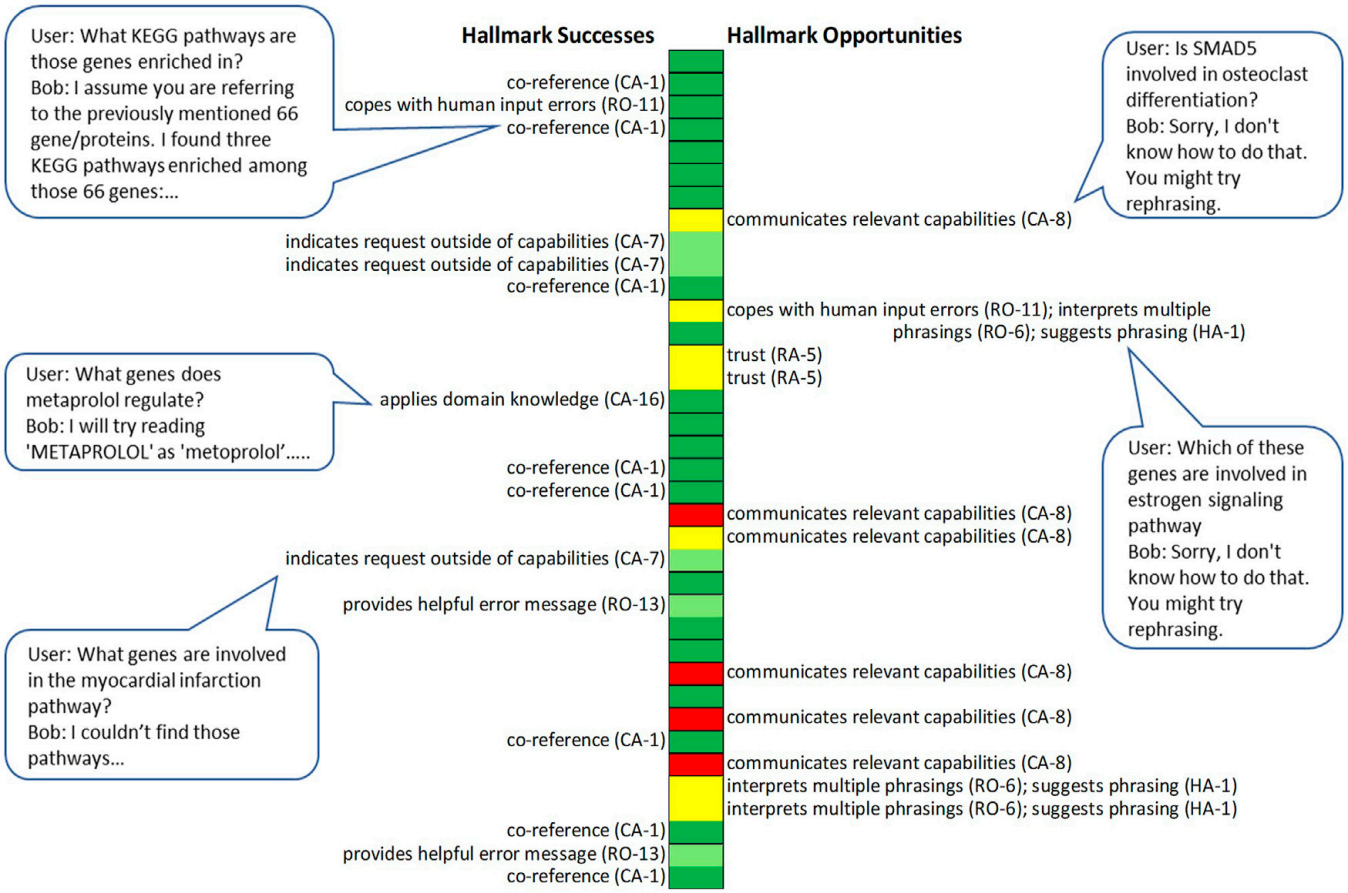
Hallmark successes and opportunities in a researcher session with Bob. Each block outlined in black marks a single or set of utterances aimed at obtaining a particular kind of information or achieving a particular action. Colors indicate the assessment of each Bob response: dark green represents appropriate biological answer or action; light green represents a helpful suggestion; yellow represents an unhelpful or misleading response for which the human partner later found a way to obtain what they were looking for; red represents an unhelpful or misleading response to a query for which the human partner never obtained an answer. Bubbles show selected single exchanges from the session.

We also used the Hallmarks to identify successes and opportunities for improving Bob in a realistic context. In 2020, the Bob development team conducted a user study in which five research biologists each interacted with Bob for about an hour. They brought and addressed their own research questions so that they had sufficient expert knowledge to readily engage, understand the meaning of Bob’s responses, and evaluate whether Bob’s contributions were useful. We then analyzed the logs and survey responses to identify *Hallmark successes*, where correct or helpful machine responses were enabled by particular Hallmarks, and *Hallmark opportunities*, where unhelpful responses could have been improved if particular Hallmarks had been achieved. We focused on the Hallmarks in the **Robustness** subcategories ***Understanding diverse communications*** and ***moving past misunderstandings***; **Context-awareness**; and **Habitability**. Figure 11 depicts Hallmark successes and opportunities identified *via* log analysis in one researcher session.

In combining all the researcher logs and survey results, we identified top Hallmark successes and opportunities based on how frequently they were associated with successes and problems and how impactful these were. Here is an example of a top Hallmark success, the specific capabilities associated with it, and their aggregate impact:

• **Hallmark: Context-awareness** CA-1: *The machine recognizes co-referring mentions of a previously mentioned entity (co-reference resolution)*
• **Capability:** Correct interpretation of “these” and “those” referring to answers from a previous query, and interpretation of pointing to lists in prior Bob responses.
• **Incidence and Impact:** All researchers extensively referenced items in prior answers. Forty-five percent of answer-yielding human utterances made use of co-reference. All human participants reported that co-reference generally worked, and one participant commented: “the follow-up feature makes it much more intuitive than manually searching through the internet.” Co-reference enabled human partners to efficiently winnow results that otherwise would have taken a long time to sort through.

Here is an example of a top Hallmark opportunity from the study (this one may be familiar to users of some current, common home smart devices):

• **Hallmark: Context-awareness** CA-8: *The machine communicates its situationally relevant capabilities to the human*.
• **Outstanding Challenge:** Human partner requests a capability closely related to one the system has, but the system performs no action and offers no suggestion. Example: Human: What genes are involved in osteoporosis? Bob: Sorry, I don’t know how to do that. You might try rephrasing.
• **Incidence & Impact:** Occurred in 5% of all exchanges in the user study, yielding unhelpful responses; four of five researchers in the user study experienced it and indicated in the survey that the computer had some problems understanding them.

In addition, the analyses identified the **Context-awareness** Hallmark CA-9: *Indicates that it doesn’t understand what a particular entity/action/word/gesture is* as a Hallmark success. Top Hallmark opportunities included further improvements in RO-6: *The machine understands multiple phrasings for similar requests* (under **Robustness**) and HA-1: *Machine offers information that helps human partners produce utterances that the machine understands* (under **Habitability**); often these were alternative potential solutions for the same problem. In addition, while **Context-awareness** Hallmark CA-1: *Machine recognizes co-referring mentions of previously mentioned entities* was a success; it also presented an opportunity; research logs indicated that it could be useful to extend this capability to include understanding a greater variety of co-reference language and allow researchers to refer to prior lists by name.

## Researcher Feedback

A survey was developed to solicit feedback from the CwC researchers on the usefulness of the Hallmarks evaluation approach used during the CwC program. The goal of the survey was to determine the effectiveness of the Hallmarks evaluation approach for the CwC program and its potential for use in future evaluations. The Hallmarks survey sent to the researchers was open for 3 weeks. Thirty-one people were sent the survey, and fifteen responded to it. All responses were collected anonymously. The survey solicited feedback using fifteen Likert statements and collected general comments and comments on each Likert statement response. The Likert statements covered four main topics: effects of the Hallmarks approach on research and system development, the fit of the Hallmarks to the research and system development, the use of the Hallmarks approach for evaluation, and the breadth of the Hallmarks approach. Overall, the researchers reported that the Hallmarks approach provided useful guidance for their research and system development, and they were mostly satisfied with the Hallmark categories and individual Hallmarks. The percentage of respondents who agreed or strongly agreed with each statement is as follows:

• The Hallmark categories^4^ helped drive my research and system development (80%)
• I have used the Hallmark categories in communicating about my research in publications/presentations (60%)
• Some of the Hallmarks aligned well with my research and system development goals (100%)
• I understood what the Hallmarks meant (93%)
• The number of Hallmarks allowed me to find ones well-suited for evaluating my system (93%)
• My team used at least one Hallmark as the basis for a metric communicated beyond the (research) program (67%)
• The Hallmarks provided me with a way to track progress towards my research and system development goals over time (66%)
• I would like to see additional Hallmarks added (7%)
• I would like to see the Hallmark list trimmed (54%)
• One or more Hallmarks led us to develop a meaningful capability that we would have not pursued otherwise (47%)
• Applying the Hallmarks helped me develop a better and more robust system (73%)
• The Hallmarks approach provides me with an effective/useful method of measuring my research and system development progress (80%)
• For measuring my research and system development process, the Hallmarks approach is superior to tracking a few metrics (53%)
• The Hallmarks approach (possibly with different categories and Hallmarks) is applicable to other research and development efforts in which I am involved (71%)
• The feedback that MITRE provided me about how well individual Hallmarks fit my work was useful to my research and system development (86%)

Full results of the survey are shown in Supplementary Figure S1.

## Literature Review

Evaluation has been a critical component of research and technology development in human-computer communication. There is a long history of formal evaluations in the speech and natural language community starting with text/message understanding (Sundheim 1991; Hirschman 1998a), natural language understanding (Guida and Mauri 1986; Paroubek, Chaudiron, and Hirschman 2007), speech recognition (Furui 2007), and transitioning to spoken language understanding (Hirschman 1998b). Each of these formal evaluations required the collection of logs of data gathered by each system about information extracted from text or speech. The collected information was compared to information that was expected in the context of the assigned task – the answer key or data – and the provided input text, input speech, and underlying dataset. These types of evaluations are called *log-based evaluations*. In this section, we look back at how formal evaluations were performed on speech and natural language systems and where those techniques may be relevant to human-computer collaborative (dialogue) systems (Terveen 1995) such as collaborative assistants. Supplementary Table 2 summarizes the different evaluation approaches discussed in this section.

Speech recognition evaluation considers recognized words in a speech segment and compares them to the actual human-transcribed words. Speech recognition evaluation metrics focus on the size of the vocabulary (represented as perplexity), isolated word error rate (based on words incorrectly recognized *versus* all words), and continuous word error rate (based on the number of substitutions, insertions, and deletions in the speech) (Furui 2007). Spoken dialogue system evaluation combines elements of text/message and speech recognition evaluations. One of the first evaluations for spoken dialogue systems was for ATIS (Air Travel Information System). It involved comparing system-extracted information with human-extracted information stored in a dataset as tuples (Price 1990; Hirschman 1998a). Hirschman described the derived evaluation metrics as sentence error (speech in, correct set of words out), spoken language understanding (speech in, database tuples out), and natural language understanding (correct transcription in, database tuples out). PARADISE (Walker et al., 1997; Walker et al., 2000) defines a general framework for evaluating spoken dialogue systems that can be applied across a broad range of spoken dialogue systems by incorporating methods from decision theory. The framework provides a “task representation that decouples what an agent needs to achieve in terms of the task requirements from how the agent carries out the task *via* dialogue” (Walker et al., 1997). The PARADISE model correlates spoken dialogue system performance with meaningful external criteria such as usability and breaks usability down into two factors-task success and dialogue cost (Walker et al., 1997). An advantage of this approach is that it allows for comparison between agents that are performing different tasks (Walker et al., 1997).

The tasks underlying these formal evaluations were primarily *closed domain*, with tight restrictions on what could be discussed in the text or speech by limiting it to a very specific domain (Deriu et al., 2021). More recently, conversational dialogue systems are being developed that involve more extended and collaborative interactions between a human and the system than in the past. These interactions can include textual or spoken exchanges as well as communication using different modalities such as gestures or facial expressions. The underlying tasks are primarily *open domain*, where what can be discussed in the text or speech is not limited in scope (Deriu et al., 2021). Context becomes increasingly important in dialogue understanding since both the linguistic and physical world can change as the conversation evolves. These complexities affect how one must conduct a formal evaluation of a dialogue system and limit the ability to automate the evaluation (Malchanau et al., 2019). Techniques for performing evaluations have evolved in recent years to include crowd-sourcing, where evaluators can take advantage of the vast access to potential users through online crowd-sourcing platforms like Amazon’s Mechanical Turk (Deriu et al., 2021). Crowd-sourcing-based evaluations can provide a lot of results in a short period of time.

The current expansion of dialogue systems has introduced collaborative assistants such as chatbots and voice-activated assistants and a need for methodologies to evaluate them. A recent study on chat-based dialogue for the PersonaChat task (See et al., 2019) used crowd-workers to chat with the system followed by answering questions focused on capturing different aspects of conversational quality. See et al. (2019) focused on four aspects they expected would improve the dialogue “*via* control (avoiding repetition, interestingness, listening, inquisitiveness), two important error classes we thought would be affected by our controls (fluency, making sense), and two overall quality measures (engagingness, humanness)” (See et al., 2019). Results of their study showed that controlling for repetition, specificity, and question-asking led to large engagingness improvements over their baseline dialogue models (See et al., 2019). They found controlling multi-turn (self) repetition is important and needs to be included with other attribute control methods, but there is no improvement by controlling response-relatedness (See et al., 2019). In particular, they found reducing repetition improves all aspects of conversational quality, increasing specificity improves interestingness and listening ability, and increasing question-asking improves inquisitiveness and interestingness over the repetition-controlled baseline (See et al., 2019). These evaluation results “show that controlling low-level attributes over multiple turns leads to improved overall quality” (See et al., 2019). These results can provide some potential guidelines for implementing better dialogues between human users and collaborative assistants.

The development of conversational dialogue agents including Apple’s Siri, Amazon’s Alexa, and Google’s Assistant has introduced new human-computer communication paradigms requiring evaluation. A recent study (Venkatesh et al., 2018) investigated multiple metrics for evaluation of conversational agents: Engagement, Coherence, Topical Metrics, User Experience (including Expectation, Behavior and Sentiment, Trust, and Visual Cues and Physicality), Domain Coverage, Conversational Depth, and Topical Diversity/Conversational Breadth. Venkatesh et al. performed an analysis of Alexa prize submissions using these metrics. Engagement was measured by the number of dialogue turns and dialogue duration (Venkatesh et al., 2018). Coherence^5^ was defined as a metric and used to evaluate dialogues: “A coherent response indicates a comprehensible and relevant response to a user’s request … we annotated hundreds of thousands of randomly selected interactions for incorrect, irrelevant or inappropriate responses. Using the annotations, we calculated the response error rate (RER) for each socialbot” (Venkatesh et al., 2018). The RER provides a measure for coherence of a dialogue. The evaluation metrics can be aggregated to provide a unified metric (Venkatesh et al., 2018). These metrics capture important characteristics of successful dialogues between human users and collaborative assistants.

Malchanau et al. (2019) examine the application of general usability metrics defined in ISO standards in the evaluation of multimodal dialogue systems. They found the ISO standards on usability and qualitative metrics for effectiveness, efficiency, and satisfaction to be very relevant when measured through results from administering their Usability Perception Questionnaire to users (Malchanau et al., 2019). Amershi et al. (2019) define a set of 18 generally applicable design guidelines for human-AI interaction that might also be useful as a basis for the evaluation of collaborative dialogue systems (Amershi et al., 2019). The developers of the conversational agents in smart devices have developed their own guidelines for good conversations based on usability requirements (e.g., Conversation Design: Speaking the Same Language^6^ and the Amazon Alexa Skills Design Guide^7^), and a set of usability heuristics for evaluating speech-based smart devices has been created. Wei and Landay (2018) developed an evaluation methodology for smart devices that use voice interfaces. The evaluation methodology defines a set of 17 design heuristics, many of which map to the Hallmarks defined in this document. Eight different evaluators performed common tasks on three smart devices. They rated the ease/difficulty of performing the tasks and identified usability issues. Wei and Landay (2018) then mapped the usability issues to their design heuristics to identify the key problems with each device (Wei and Landay 2018). The results were assembled to identify the key problems and heuristics that were violated. Evaluators reported that the heuristics enabled them to evaluate more thoroughly. Zwakman et al. (2021) look at usability evaluations of AI-based voice assistants with an emphasis on Amazon Alexa (Zwakman et al., 2021). The authors extend the use of System Usability Scale (SUS), often used in evaluating the usability of Graphical User Interfaces (GUI), to a Voice Usability Scale (VUS) for voice assistants. Zwakman et al. (2021) propose usability dimensions that align with many of the Hallmarks. They assess voice assistants across six usability dimensions: General usability (general thoughts about using the voice assistant), Affective (the psychological and feelings of the user), Recognition and visibility (the intuitiveness of the interactions with the voice assistant), Pragmatic (ability to accomplish tasks), Errors and frustration (prevention and handling of errors), and Guidance and help (guiding users on the use of the voice assistant to accomplish a task) (Zwakman et al., 2021).

This heuristic-based approach to evaluating the conversational agents in smart devices evolves from a more general approach to evaluating the usability of user interfaces – Heuristic Evaluation for User Interface (Molich and Nielsen 1990). A heuristic evaluation of a user interface involves an informal approach to usability analysis through the inspection of a user interface to identify potential usability problems in its design^8^. The method was developed by Rolf Molich and Jakob Nielsen (Molich and Nielsen 1990; Nielsen and Molich 1990) and refined by Nielsen and Mack (1994) into a set of ten usability heuristics.

Specific metrics of good communication have evolved over the course of research on human communication and conversational systems that influenced the Hallmarks for human-computer collaborative systems discussed in this study. Watt (1968) introduced the notion of a habitable language in which humans can easily learn to express themselves without straying out of the language’s boundaries and argued that any practical human-computer interface must be a habitable subset of the English language (Watt 1968). The Grice Maxims of effective conversation (Grice 1989) focus on the ways humans communicate to be understood and most effective in conveying their intentions: Maxim of Quantity (be informative but not more than necessary), Maxim of Quality (be truthful), Maxim of Relation (be relevant), and Maxim of Manner (be clear; be brief).

## Discussion

The *Hallmarks of Human-Machine Collaboration* aims to provide a flexible framework for the evaluation of a wide range of difficult-to-evaluate creative collaborative assistants that is both evaluative and diagnostic, exposing areas of strength and opportunities for improvement. In addition to assessment, it is intended to be used to drive research directions, improve system development, and gauge success at achieving research goals.

These Hallmarks evolved over the course of several years of use, as we learned that excelling in one area might cause problems in another and attempted to ensure that both sides of such trade-offs were represented. For example*,* a system that permitted only a very small number of well-defined inputs could be extremely **Habitable** but would be lacking in the ability to understand diverse communications under **Robustness** (and likewise, a system accepting too broad a range of communications might lack sufficient guidance for the human partner to be habitable). Similarly, a system with a computationally expensive means of representing and using context might show great **Context-awareness** but be so slow as to fail to be **Consistently Engaging** to the human partner. **Habitability** and **Consistent Human Engagement** are Key Properties that were added after the initial version of the Hallmarks were shared with CwC research teams, in large part to help ensure that Key Properties were not achieved in ways that hurt other aspects of the collaboration.

The Hallmarks-based evaluation approach described in this document builds on prior evaluation methodologies. It has some similarities to the approach followed for the Heuristic Evaluation for User Interface methodology, and there is some overlap between our Key Properties and Hallmarks and the lists of Heuristics in other studies (Molich and Nielsen 1990; Wei and Landay 2018; Zwakman et al., 2021). But just as each of these sets of Heuristics differs from the others due to their focus on different types of human-machine interactions (e.g., speech-based smart devices, user interfaces in general), our Key Properties and Hallmarks also differ from each of these due to our particular focus on machines as collaborative partners on open-ended, creative activities.

One of the things that appear to be unique in our framework is making the notion of ***worthwhile collaboration*** explicit. Hallmarks SC-3, SC-4, and SC-5 (see *Key Properties of Human-Machine Collaboration* section) require working with the machine partner to result in a better product, or an easier or more enjoyable process. We introduce two more novel subcategories, inspired by the goal first expressed by Licklider (1960) that the human and computer should collaborate in much the same way that a human would collaborate with a human colleague. The first is the ***appropriate and collaborative contributions*** subcategory of our **Mutual Contribution of Meaningful Content Key Property**:

MC-6. The machine makes meaningful contributions to the interaction
MC-7. The machine enables the human to make meaningful contributions to the interaction
MC-8. Partners negotiate or collaboratively shape goals or approaches

This set gets at the collaborative nature of the interaction that we aim for. The second is the ***composition*** subcategory under **Use of Elementary Concepts to Teach and Learn New Concepts**:

EC-2. Humans can teach the machine a new concept by presenting it as a composition of more elementary known concepts
EC-3. The machine can learn (or infer) the meaning of a new word or concept without explicit human instruction
EC-4. The machine introduces or explains a new concept by presenting it as a composition of more elementary known concepts

The need for the collaborative machine partner to teach and learn new concepts is critical to emulating a human-human collaboration, where partners must frequently teach one another new concepts. Although context-awareness is hardly unique to our framework, the set of 18 Hallmarks that we have collected under the **Context-awareness** Key Property (across four subcategories) represent a more complete collection than we’ve found in any other evaluation methodology.

There are some heuristics in other concurrently developed lists that in retrospect could be useful additions to the Hallmarks (Wei and Landay 2018; See et al., 2019; Amershi et al., 2019; Zwakman et al., 2021). In particular, Wei and Landay (2018) included *Give the agent a persona, Make the system status clear, and Confirm input intelligently*. We observed that developers of multiple systems that we evaluated nevertheless incorporated methods to meet these heuristics, such as through embodied forms and friendly names for the machine partners, by having the machine indicate when something was still running and taking some time, and by the machine stating how it interpreted a human’s utterance. These additions did seem to substantially improve the human-machine collaboration in these systems.

Due to the subjective nature of a Hallmarks-based assessment, there can be variability in assessments performed by different evaluators. This challenge can be mitigated by asking evaluators to justify their evaluations and by having multiple evaluators apply the Hallmarks to a particular human-computer collaborative system and comparing their assessments. Depending on the needs and goals of the evaluation, this can either be used to develop detailed assessment guidelines for a Hallmark in a given context to drive evaluators toward more consistent assessments or used to provide a potentially more robust range of judgments.

While assessment using the Hallmarks can help track successes and expose shortcomings of individual systems, given the highly subjective nature of these assessments, and the variety of different ways that different systems might demonstrate progress toward the eight Key Properties, it is not effective at or intended for comparisons across systems. Similarly, this methodology may not expose the root cause behind a Hallmark not being met. In this case, finding or developing a more narrowly focused metric-based approach that can delve deeper into a particular aspect of the implementation may be warranted.

Hallmarks-based evaluation is not intended to replace, but instead to complement, existing approaches. If there are appropriate metrics to evaluate parts of a collaborative machine partner, these should still be used. For example, the Sentence Error Rate metric from speech recognition can tell us how well the speech recognizer is correctly mapping a spoken utterance into text, which can affect Hallmarks such as RO-5: *The human’s communication is correctly interpreted by the machine*. However, it is important to ensure that the metrics are applied to measure something genuinely important to the goals of the system. It may be tempting to use a metric such as task completion time, but in many collaborative human-machine partnerships, speedy completion is not a high priority. In a creative partnership, the quality of the end product may be more important than how long it took to complete. And in some cases, it is the journey and not the destination that is important, such as when jamming with an Interactive Jazz system or free-form collaborative building with a Blocks World system.

The CwC research teams were given Hallmark assessments of their systems at multiple points during the research program. The teams often responded to these assessments by making it a priority to address failed or missing Hallmarks most relevant to their system and research goals. The SIFT team working on the Bob molecular biology collaborator published results from one of the targeted tests the MITRE T&E team performed on their system (Burstein et al., 2020). They noted that some of the tests relied on capabilities that they had not yet implemented and indicated that these capabilities became priorities following the testing.

Many CwC research teams applied the Hallmarks on their own to help them plan next steps in their development process. In informal communication with the research teams, we frequently heard that our definition and use of the Key Properties and Hallmarks for program evaluation in the CwC program helped guide their research and development by articulating priorities. The result of using the Hallmark methodology was new or revised research goals and improvement of systems over the course of the research program. The Hallmarks guided feature development and interface design and provided a common framework for discussing work with other research teams. Systems got better while at the same time addressing important overall goals of the research program.

Some teams extended or modified the MITRE Hallmarks to best fit their research goals. The Brandeis Diana team published an evaluation framework (Krishnaswamy et al., 2020) for their specific use case – multimodal interaction with an embodied virtual agent – that built upon an earlier version of the Hallmarks focusing on a use-case-specific subset motivated by past communication research (Hobbs and Evans 1980; Ligozat 1993; Arbib and Rizzolatti 1996; Zimmermann and Freksa 1996; Wooldridge and Lomuscio 1999; Asher and Gillies 2003; Arbib 2008; Johnston 2009). That team also told us, “The introduction of the Hallmarks into the program was a seminal development in Brandeis’s creation of computational ‘common ground’ as a motivating factor and underpinning of our use of simulation in our approach to CwC.” (Krishnaswamy and Pustejovsky, personal communication, 2019).

We conducted an anonymous survey of CwC researchers to get feedback on the Hallmarks assessments, and the results were largely positive as described in *Researcher Feedback* section. Most performers (80%) were positively aligned with the statement, “The Hallmark categories helped drive my research and system development.” The only strongly negative response (67% selected *disagree*) was to the item, “I would like to see additional Hallmarks added.” Most respondents (93%) were positively aligned with the statement, “I understood what the Hallmarks meant.” Informal survey feedback from the teams was mostly positive about the use of Hallmarks for evaluation. The Colorado State University and University of Florida Diana team told us, “The Hallmarks are extremely helpful because they concisely express basic aspects of human-computer communication largely missing from current agents” (Ross Beveridge and Jaime Ruiz, personal communication, 2019).

The primary complaint was that the large number of Hallmarks across all the Key Properties was overwhelming. We provide such a large number of Hallmarks in order to ensure coverage of important features needed for true human-computer collaboration, while also providing sufficient flexibility for different types of systems to demonstrate progress toward a Property in different ways. In the context of the CwC program, we expected systems to exhibit a selection of Hallmarks that they identified as being aligned with the research goals of their projects; no system was expected to “check off” every Hallmark. While research teams indicated that they did understand this, they still felt that it was a lot to consider.

The Hallmarks approach can be applied broadly across disparate use cases as illustrated by the breadth of use cases and examples described above. It allows system developers or external evaluators to track progress toward achieving multiple important characteristics of computer systems that can collaborate as partners with humans on complex, open-ended activities.

While we have demonstrated the utility of this evaluation approach across a variety of use cases, we have not formally validated the framework. It remains for future work to validate the clarity of the framework by comparing multiple assessments of the same system by different evaluators, to ensure that it can be consistently applied. In addition, the general applicability of this framework could be assessed by having independent evaluators apply the Hallmarks to additional collaborative machine partner systems created in other contexts and provide feedback about the Hallmarks’ ease of use, comprehensiveness, and applicability for the systems.

## Conclusion

Creating collaborative machine partners for society means that a collaborative assistant has to interact in more complex and natural ways with humans (e.g., multimodal) and provide substantive contributions to the mutual interaction that helps humans achieve important goals that benefit society. Evaluating such collaborative machine partners requires much more than traditional metric-based approaches to be able to cover such broad interactions with humans. The Hallmarks approach to evaluation, coupled with more traditional metrics-based approaches, can reveal important areas of success in providing true collaborative machine partners for society while helping researchers and developers address areas of weakness revealed in an evaluation. While future work is indicated to further validate the framework, our use of the Hallmark approach in the CwC program illustrated its broad applicability and usefulness across diverse use cases in guiding the research teams in achieving their research goals while focusing on important desired characteristics of collaborative systems.

## Data Availability

The original contributions presented in the study are included in the article/Supplementary Material; further inquiries can be directed to the corresponding author.

## Footnotes

1 This Key Property has been modified somewhat from the version presented in (Kozierok et al., 2021) where the property was tied to a DARPA Communicating with Computers program-specific notion of “Elementary Composable Ideas.”

2 A version of this goal is expressed in the description of the DARPA Communicating with Computers program (https://www.darpa.mil/program/communicating-with-computers).

3
https://demo.musicaresearch.org/.

4 Note that “Hallmark categories” was an earlier phrasing used to refer to what we now call “Key Properties.”

5 The metric Coherence overlaps with a portion of the **Context-awareness** Key Property defined in this paper.

6
https://design.google/library/conversation-design-speaking-same-language/.

7
https://developer.amazon.com/en-US/docs/alexa/alexa-design/get-started.html.

8
https://en.wikipedia.org/w/index.php?title=Heuristic_evaluation.
