# Supplementary material for: Assessing Open-Ended Human-Computer Collaboration Systems: Applying a Hallmarks Approach
Source: Front Artif Intell. 2021 Oct 18;4:670009. doi: 10.3389/frai.2021.670009 (PMC8561722; doi:10.3389/frai.2021.670009)
Supplement: Supplementary file 1 [file Table1.docx]

Supplementary Material

**Supplementary Table 1**: A listing of the full set of Hallmarks for each of the Key Properties and subcategories. For those where we judged that the human partner’s feedback is necessary to fully assess achievement of the Hallmark, we have provided standard Likert items to be used as survey questions in user surveys following interaction with the system. Note that in some cases the survey question gets at only part of the Hallmark, where some aspect of the Hallmark can be directly observed. In a few cases the polarity of the Likert item is the opposite of the Hallmark. For example, the item “The system behaved in unexpectedly odd ways” probes for failures of Hallmark HE-4: *The machine communicates without creating undue distraction.*

| **Key** **Properties** | **Subcategories** | **Hallmarks** | **Survey Questions** |
| --- | --- | --- | --- |
| **Successful Collaboration**  Satisfying creative collaborations can take place in which machines are not merely receivers of instructions but are full collaborators | ***Efficient, collaborative project completion*** | SC-1. The human-machine team completes projects | I got exactly the result I wanted. |
|  |  |  | I was satisfied with the results I achieved with the system. |
|  |  | SC-2. Task completion is efficient | It was easy to interact with the system to get a good outcome. |
|  | ***Worthwhile collaboration*** | SC-3. It’s easier to do the activity together than alone |  |
|  |  | SC-4. Doing the activity together results in a more interesting, creative, or otherwise better product |  |
|  |  | SC-5. It is more enjoyable to do the activity together than alone |  |
|  | ***Human satisfaction*** | SC-6. Overall, the human is satisfied with the collaboration | Overall, I was satisfied with my interaction with the system. |
|  |  | SC-7. Humans want to interact further with the machine | I would like to engage with the system again. |
| **Robustness**  Efficient task-based interaction proceeds smoothly as long as the human wants to, without resets | ***Software reliability and consistency*** | RO-1. The interaction proceeds without the need for resets (no crashes/hangs) |  |
|  |  | RO-2. All expected capabilities are online and working as expected |  |
|  |  | RO-3. The machine produces consistent content | The machine provided inconsistent content across turns. |
|  |  | RO-4. The machine responds with appropriate actions |  |
|  | ***Ability of human and machine to understand diverse communications*** | RO-5. The human's communication is correctly interpreted by the machine | The system responded appropriately to my communications (speech, gesture, typing, etc.). |
|  |  | RO-6. The machine handles multiple phrasings or forms for similar requests |  |
|  |  | RO-7. The machine's communication is readily interpreted by the human | It was easy to understand the system's communications. |
|  |  | RO-8. The machine communicates effectively via multiple modalities (e.g., speech, gesture, text, audio, facial expressions), as appropriate | The system communicated effectively by [multiple modalities, system specific]. |
|  |  | RO-9. The machine correctly interprets multiple communication modalities | The system correctly interpreted my communication by both [multiple modalities, system specific]. |
|  |  | RO-10. The set of inputs the machine can interpret is enough to support the full functionality of the system | I was able to express what I wanted to the system. |
|  | ***Ability of the machine to move the conversation forward past misunderstandings*** | RO-11. The machine copes with errors in the human's input |  |
|  |  | RO-12. The machine repairs interactions and/or supports human repair |  |
|  |  | RO-13. The machine provides helpful/actionable error messages | The system's error messages were helpful. |
|  |  | RO-14. The machine asks clarifying questions as needed |  |
|  |  | RO-15. The machine responds appropriately to humans' requests for clarification |  |
| **Mutual Contribution of Meaningful Content**  Each participant makes meaningful contributions to the session, and either party can take or cede initiative | ***Machine’s knowledge of when to act and how much to contribute*** | MC-1. Partners each take multiple turns in the interaction |  |
|  |  | MC-2. Each partner knows when to communicate and/or take actions | I knew when to take my turn. |
|  |  | MC-3. Machine responses are of an appropriate length and level of detail | The system responses were of an appropriate length and level of detail. |
|  |  | MC-4. The machine takes initiative when appropriate |  |
|  |  | MC-5. If the human grants autonomy, the machine responds appropriately |  |
|  | ***Appropriate and collaborative contributions*** | MC-6. The machine makes meaningful contributions to the interaction | I felt that the system and I were working collaboratively. |
|  |  | MC-7. The machine enables the human to make meaningful contributions to the interaction | The system enabled me to make meaningful contributions to the interaction. |
|  |  | MC-8. Partners negotiate or collaboratively shape goals or approaches | The machine and I collaborated to come up with our goals or approaches. |
| **Consistent Human Engagement**  Humans find engaging with machine comfortable, useful, fun, inspiring, and/or rewarding | ***Comfortable interaction*** | HE-1. Human partners can communicate successfully in a way that is comfortable | I was able to communicate successfully in a way that was comfortable. |
|  |  | HE-2. The human is satisfied with the pacing/tempo of the interaction | I was satisfied with the pacing of the interaction. |
|  |  | HE-3. The human is satisfied with how things progress during the interaction | I was satisfied with the path of the interaction. |
|  |  | HE-4. The machine communicates without creating undue distraction | The system behaved in unexpectedly odd ways. |
|  | ***Machine’s ability to evoke and inspire*** | HE-5. The machine produces content that is interesting, novel, useful, and/or creative | The system produced content that was interesting, novel, useful, and/or creative. |
|  |  | HE-6. Machine inspires new ideas in the human partner | The system helped me come up with new ideas. |
|  |  | HE-7. The machine evokes emotional responses (other than frustration) | I was pleasantly surprised by something the system contributed. |
| **Context-awareness**  Both partners can communicate efficiently by referencing and understanding contexts, including the linguistic, conversational, and deictic context, task context, goal context, self-knowledge, the partner's abilities, and world/domain knowledge | ***Linguistic and/or deictic context-awareness*** | CA-1. The machine recognizes co-referring mentions of previously mentioned entities (co-reference resolution) |  |
|  |  | CA-2. The machine recognizes references to previously created units or sub-parts |  |
|  |  | CA-3. The machine generates references to previously created units or sub-parts |  |
|  |  | CA-4. The machine correctly interprets a term defined by a human partner |  |
|  |  | CA-5. The machine uses a term defined by a human partner | The system correctly used a term that I introduced. |
|  |  | CA-6. The machine correctly interprets and correctly uses deictic references (i.e., references situated in time and/or place, such as by pointing) |  |
|  | ***Pragmatic context-awareness*** | CA-7. The machine indicates when a requested action is outside of its capabilities | I understand the capabilities and limitations of the system. |
|  |  | CA-8. The machine communicates its situationally relevant capabilities to the human | The system helped me understand its capabilities. |
|  |  | CA-9. The machine indicates that it doesn't understand what a particular entity/action/word/ gesture is when appropriate | The system knows what it doesn't know. |
|  |  | CA-10. The machine maps its interpretation to its appropriate capabilities |  |
|  |  | CA-11. The machine tailors responses to the human partner’s apparent capabilities |  |
|  |  | CA-12. The machine tailors responses to the human partner's stated or implied goals |  |
|  |  | CA-13. Human partners tailor responses to machine's stated or implied goals |  |
|  | ***Situational context-awareness*** | CA-14. The machine responds appropriately to human references and actions in the context of the evolving situation (includes anything built and pieces available) | The system followed what I talked about across multiple turns. |
|  |  | CA-15. The machine's contributions to the interaction are consistent, relevant, and build from turn to turn (i.e. are coherent) | The system seemed to be aware of its previous actions. |
|  | ***Appropriate use of world/domain knowledge*** | CA-16. The machine applies world/domain knowledge as required | The system appeared to apply general world/domain knowledge relevant to the task. |
|  |  | CA-17. Machine contributions are generally consistent with common sense |  |
|  |  | CA-18. Machine demonstrates understanding of a broad range of vocabulary |  |
| **Provision of Rationale**  The machine can expose its reasoning, sources, and methods | ***Logging*** | RA-1. The machine can provide a human-interpretable output (trace), showing processes implemented by the computer sufficient for evaluators' use |  |
|  | ***Ability to explain rationale*** | RA-2. The machine provides information about the sources and methods it used to generate a response or action |  |
|  |  | RA-3. The machine provides reasons for its responses and actions that make sense to the human partner | The system provided reasons for its responses and actions that made sense to me. |
|  |  | RA-4. The machine answers questions about its reasoning, sources, and/or methods |  |
|  | ***Human’s trust is appropriately calibrated*** | RA-5. The human partner knows when to trust the machine's responses and actions | I believe the system is a competent performer. |
|  |  |  | I believe the system is a competent performer. |
| **Habitability**  Humans easily learn to use language and/or gestures that the machine can correctly interpret and act upon | ***Shaping*** | HA-1. The machine offers information that helps human partners produce utterances and/or gestures that the machine understands and are consistent with its capabilities | The system helped me to communicate successfully. |
|  |  | HA-2. The machine offers information that helps human partners produce utterances and/or gestures that are consistent with the partners' goals and move the collaborative effort forward |  |
|  |  | HA-3. The machine models appropriate use by generating utterances and/or gestures that it can interpret | When I used the same language/gestures that I saw the system use, the system understood me. |
|  |  | HA-4. The machine answers questions about its capabilities |  |
|  |  | HA-5. The machine uses and understands language consistently (across capabilities) |  |
|  | ***Learnability*** | HA-6. Human partners are able to quickly learn to use the system effectively | It was easy to figure out how to communicate effectively with the system. |
|  |  | HA-7. The collaborative effort moves forward with minimal need for repeats or digressions |  |
|  |  | HA-8. The number of repair sub-dialogues decreases over time for each human partner |  |
| **Use of Elementary Concepts to Teach and Learn New Concepts**  Uses and composes elements of a set of elementary concepts to represent more complex concepts | ***Representation*** | EC-1. The machine can represent concepts as a composition of more elementary concepts |  |
|  | ***Composition*** | EC-2. Humans can teach the machine a new concept by presenting it as a composition of more elementary known concepts |  |
|  |  | EC-3. The machine can learn (or infer) the meaning of a new word or concept without explicit human instruction |  |
|  |  | EC-4. The machine introduces or explains a concept new to the human partner by presenting it as a composition of more elementary known concepts |  |
