# Supplementary material for: Assessing Open-Ended Human-Computer Collaboration Systems: Applying a Hallmarks Approach
Source: Front Artif Intell. 2021 Oct 18;4:670009. doi: 10.3389/frai.2021.670009 (PMC8561722; doi:10.3389/frai.2021.670009)
Supplement: Supplementary file 2 [file Table2.docx]

Supplementary Material

**Supplementary Table 2.** Summary of Evaluation Approaches: We compare many of the evaluation approaches described in the Literature Review on the basis of target system type, evaluation type, a brief description of the technique, and a list of metrics or criteria.

| **Target** | **Types** | **Technique** | **Metrics/Criteria** |
| --- | --- | --- | --- |
| Speech Recognition  (Speech) | Closed Domain    Log-based | Compare recognized words to human-transcribed words (ground truth) | Precision  Recall  Perplexity  Word Error Rate  Continuous Word Error Rate  (*Furui 2007*) |
| Spoken dialogue  (Speech)    Task-Oriented | Closed Domain    Log-based | Measure understanding and task performance when couple speech recognition with language understanding  (ground truth) | Sentence Error Rate (speech in, correct set of words out)  Spoken Language Understanding (speech in, database tuples out)  Natural Language Understanding (correct transcription in, database tuples out)  (*Hirschman, 1998*) |
| Spoken dialogue  (Speech)    Task-Oriented | Closed Domain    Decision theory based    Log-based | Decouple what an agent needs to accomplish a task from how the task is carried out via (spoken) dialogue while maximizing  user satisfaction and using task success and various interaction costs as predictors of user satisfaction *(Walker et al. 1998, pp. 271-272)* | Dialogue Efficiency Metrics  **–** Total elapsed time, Time on task, System turns, User turns, Turns on task  **–** Time per turn for each system module  Dialogue Quality Metrics  **–** Word error rate, Reprompts, Error messages, Help messages, Response latency.  **–** Mean word error rate, Reprompt %, Mean response latency, Variance response latency, Help %  Task Success Metrics – Perceived task completion, Objective task completion  User Satisfaction – Sum of TTS performance, Task ease, User expertise, Expected behavior, Future use.  (*Walker et al. 1997*)  (*Walker, Hirschman, and Aberdeen 2000*) |
| Multimodal Dialogue System    Chatbots | Open Domain    Survey-based | Crowd sourcing  Focus on capturing different aspects of conversational quality from responses to questions in crowd-worker surveys | Control (avoiding repetition, interestingness, listening, inquisitiveness)  Error classes affected by the controls (fluency, making sense)  Overall quality measures (engagingness, humanness)  *(See et al. 2019, pg. 1708)* |
| Multimodal Dialogue System    Conversational dialogue agents | Open Domain    Log-based | Analysis of logs and annotated responses | Engagement, Coherence, Topical Metrics  User Experience (including Expectation, Behavior and Sentiment, Trust, and Visual Cues and Physicality)  Domain Coverage, Conversational Depth, and Topical Diversity/Conversational Breadth  *(Venkatesh et al., 2018)* |
| Multimodal (Collaborative) Dialogue System    Conversational dialogue agents | Open Domain    Usability-based    Survey-based | Measure general usability metrics defined in ISO standards in the evaluation of multimodal dialogue systems using a Usability Perception Questionnaire given to users *(Malchanau et al., 2019)*    Determine if a set of 18 generally applicable design guidelines for human-AI interaction apply to a collaborative dialogue system *(Amershi et al., 2019)*    Determine if a set of usability heuristics apply to a collaborative dialogue system *(Wei et al., 2018)* | General usability metrics  *(Malchanau et al., 2019)*  *(Amershi et al., 2019)*  *(Wei et al., 2018)* |
| Multimodal (Collaborative) Dialogue System    General human-computer collaborative creation tasks | Open Domain    Log-based    Survey-based | Check for properties of successful dialogue systems and instances of those properties that can be observed or measured by (human) evaluators or as determined from surveys of human partners | - - - 1. Successful Collaboration       2. Robustness       3. Mutual Contribution of Meaningful Content       4. Consistent Human Engagement       5. Context-awareness       6. Provision of Rational       7. Habitability       8. Use of Elementary Concepts to Teach and Learn New Concepts   *(Kozierok et al., 2021)* |
