# Supplementary figures and images for: Assessing Open-Ended Human-Computer Collaboration Systems: Applying a Hallmarks Approach
Source: Front Artif Intell. 2021 Oct 18;4:670009. doi: 10.3389/frai.2021.670009 (PMC8561722; doi:10.3389/frai.2021.670009)

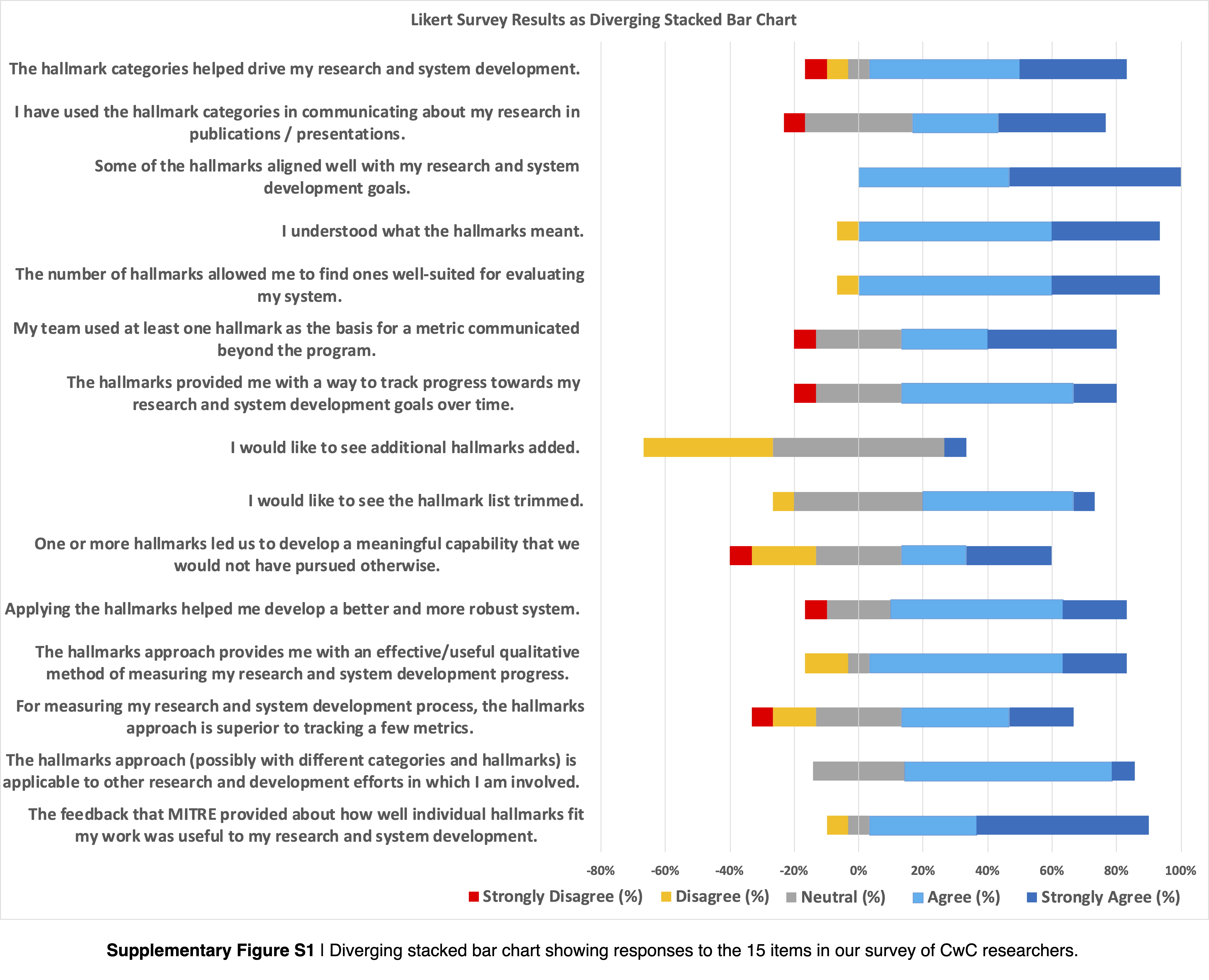

Supplement: Supplementary file 3 [file Image1.jpg]
